# The evolutionary conservation of the A Disintegrin-like and Metalloproteinase domain with Thrombospondin-1 motif metzincins across vertebrate species and their expression in teleost zebrafish

**DOI:** 10.1186/s12862-015-0281-9

**Published:** 2015-02-15

**Authors:** Frédéric G Brunet, Fiona W Fraser, Marley J Binder, Adam D Smith, Christopher Kintakas, Carolyn M Dancevic, Alister C Ward, Daniel R McCulloch

**Affiliations:** Institut de Génomique Fonctionnelle de Lyon, Université de Lyon, Université Lyon 1, CNRS, Ecole Normale Supérieure de Lyon, 46, allée d’Italie, 69364 Lyon cedex 07, France; School of Medicine, Deakin University, Geelong, VIC 3216 Australia; Molecular and Medical Research Strategic Research Centre, Deakin University, Geelong, VIC 3216 Australia

**Keywords:** ADAMTS, Evolution, Whole genome duplications, Vertebrata, Metazoa, Zebrafish, Development, Gene expression

## Abstract

**Background:**

The *A D*isintegrin-like and *M*etalloproteinase domain with *T*hrombo*s*pondin-1 motifs (ADAMTS) enzymes comprise 19 mammalian zinc-dependent metalloproteinases (metzincins) with homologues in a wide range of invertebrates. ADAMTS enzymes have a broad range of functions in development and diseases due to their extracellular matrix remodelling activity. Here, we report a detailed characterisation of their evolutionary conservation across vertebrates.

**Results:**

Using bioinformatics complemented with *de novo* sequencing, gene sequences for ADAMTS enzymes were obtained from a variety of organisms. Detailed evolutionary analyses revealed a high level of conservation across vertebrates with evidence of *ADAMTS* gene expansion during two rounds of whole genome duplication (WGD) in vertebrates, while tandem duplication events and gene loss were also apparent. However, the additional round of teleost-specific WGD did not have a significant effect on *ADAMTS* gene family members suggesting their conserved roles have remained constant in teleost fish. Quantitative reverse-transcriptase polymerase chain reaction analysis revealed dynamic expression of *adamts* genes throughout zebrafish embryonic development reflecting the key conserved roles they play in vertebrate embryogenesis. Notably, several *adamts* mRNAs were maternally expressed with a dramatic increase in mRNA levels coinciding with zygotic expression and organogenesis. Broad *adamts* mRNA expression was also demonstrated in several adult organs indicating potential roles in adult homeostasis.

**Conclusions:**

Our data highlight the evolution of the *ADAMTS* gene family through duplication processes across metazoans supplemented by a burst of amplification through vertebrate WGD events. It also strongly posits the zebrafish as a potential model species to further elucidate the function of ADAMTS enzymes during vertebrate development.

**Electronic supplementary material:**

The online version of this article (doi:10.1186/s12862-015-0281-9) contains supplementary material, which is available to authorized users.

## Background

Metzincins are a superfamily of zinc-dependent metalloproteinases that include the matrix metalloproteinases (MMPs), the A Disintegrin and Metalloproteinase Domain (ADAMs) and the A Disintegrin-like and Metalloproteinase Domain with Thrombospondin-1 motifs (ADAMTS) enzyme families. Metzincins are represented across prokaryotes and unicellular and multicellular eukaryotes [1]. All metzincins share the zinc-binding catalytic motif consensus sequence HEXXHXXGXX (H/D), whereby the three histidines (or an aspartic acid in the third position) coordinate the binding of a zinc molecule, the glutamic acid residue facilitates general acid–base catalysis and the small glycine residue allows for steric flexibility within the catalytic motif [1-3]. The first role for a metzincin was discovered while examining mechanisms of morphogenesis in tadpoles where collagenase (MMP) activity was found to contribute to the tail fin resorption [4] but they are now known to contribute to a vast array of biological processes [5].

The *ADAMTS* genes encode secreted enzymes that remodel the extracellular matrix (ECM); they play roles in cell-cell interactions such as cell signalling and fusion, as well as developmental morphogenesis in mammals and annelids [6-8]. There are 19 *ADAMTS* genes in human, designated *ADAMTS1* through *ADAMTS20*, where *ADAMTS5* and *ADAMTS11* represent the same gene [9-11]. The functional subdivisions of the 19 members are based on common affinities towards preferred substrates. The evolution of several *ADAMTS* genes has previously been reported across various species including the fugu, the urochordate *Ciona intestinalis*, and the invertebrates *Drosophila melanogaster* and *Caenorhabditis elegans* [12-14]. These studies have underscored the importance of ADAMTS enzymes throughout vertebrate evolution including the rapid expansion of this gene family concomitant with the emergence of chordates and vertebrates [12-14]. However, in light of the rapid advancement of sequences now available across vertebrates and beyond, an up-to-date analysis of the evolution of this gene family is now required.

Moreover, the complexity of understanding the cooperative ADAMTS biology in rodent models of development [8] means additional vertebrate models are required to further elucidate ADAMTS biology during embryogenesis. Zebrafish has emerged as a popular model to study vertebrate embryonic development with well conserved gastrulation, somitogenesis and organogenesis [15], which generates a body plan showing strong parallels to rodents and human [16].

In this current study, we undertook a detailed analysis of the evolutionary history of *ADAMTS* through the three major rounds of whole genome duplications (WGD) across vertebrate species using up-to-date genomic DNA databases supplemented with additional sequences from other repositories. We also examined the expression pattern of zebrafish *adamts* genes in the developing embryo and adult organs. Our analyses highlight key events in *ADAMTS* gene evolution, providing insight into their roles throughout the emergence, and then divergence of vertebrates. Collectively, our study highlights the evolution of the metzincin-superfamily of zinc-peptidases and conservation of ADAMTS protein sequences, and suggests the zebrafish as an attractive model to dissect ADAMTS biology throughout vertebrate development, with potential applications in pathological contexts.

## Results

**Figure 1:**
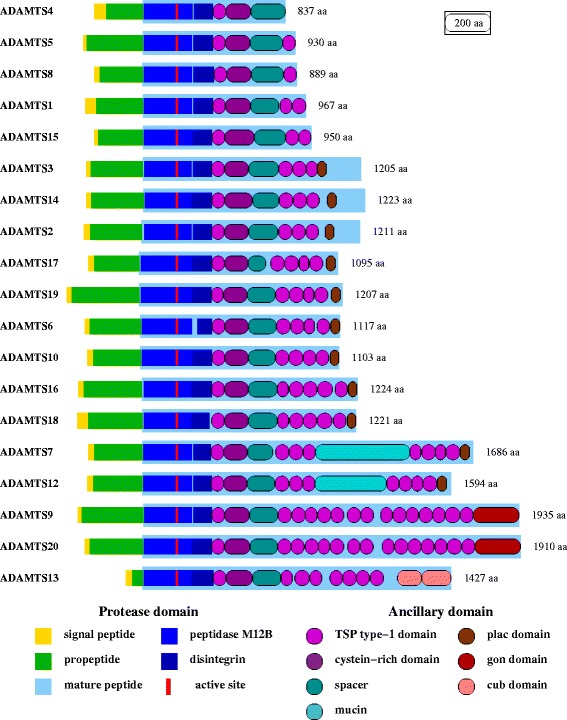
**Structural relationships amongst ADAMTS proteins** ***.*** All ADAMTS proteins share a common protease domain including the peptidase M12B and its active site, and a disintegrin-like, thrombospondin (TSP) type-1 and cysteine-rich domain. Ancillary (C-terminal) domains are variable among the genes as indicated.

The initial aim of this study was to understand the evolution of *ADAMTS* genes through the 3 whole genome duplication (WGD) events in vertebrates [17-20]. ADAMTS proteins share a common structure with distinct modules, including a propeptide region, a metallopeptidase M12B domain, a disintegrin-like and a thrombospondin (TSP) type-1 domain followed by a spacer (Figure 1). With the exception of ADAMTS4, which lacks additional C-terminal domains, all other members contain from 1 up to 14 additional TSP type-1 domains. These can form the C-terminus for ADAMTS1, −5, −8 and −15 or that can be in addition to other domains, those being PLAC for 11 ADAMTS (ADAMTS2, −3, −6, −7, −10, −12, −14, −16, −17, −18 and −19), a GON-1 domain for ADAMTS9 and −20, or a CUB domain for ADAMTS13 (Figure 1).

**Figure 2:**
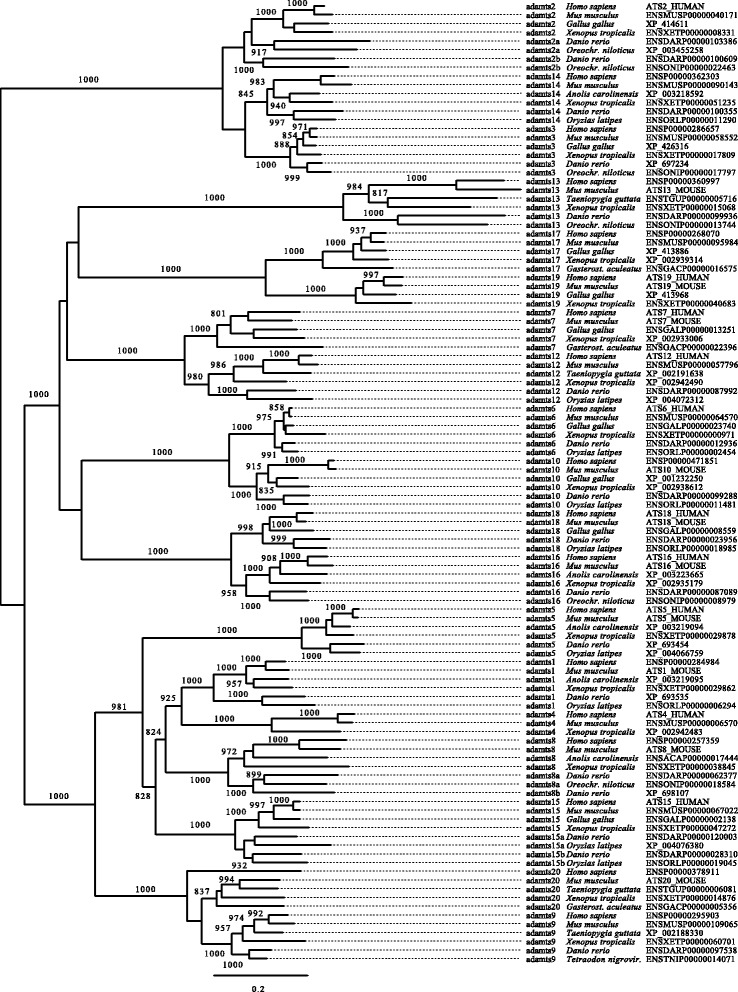
**Phylogenetic analysis of proteins of the adamts family in vertebrate species.** Only full and best aligned sequences were analyzed using maximum likelihood (ML). Branch values show the bootstrap replicates. The leaflet version of this tree is used in the central ML tree of Figure 3.

**Figure 3:**
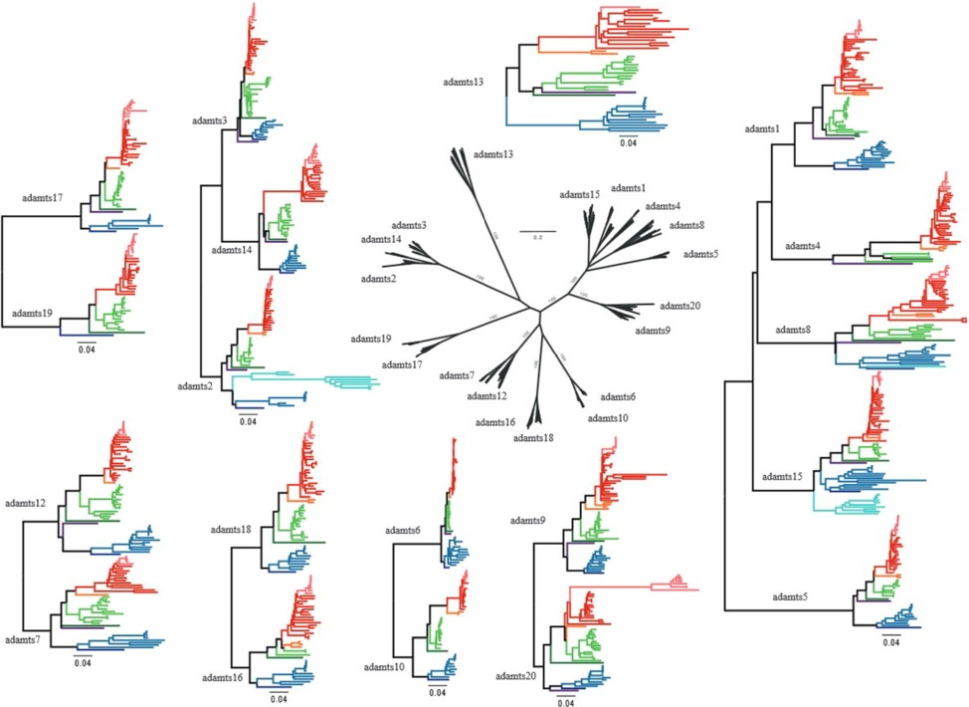
**Phylogenetic analysis of ADAMTS proteins.** Phylogenetic analyses by maximum likelihood (ML) of all proteins of the ADAMTS family based on the shared structures, which are the metallopeptidase M12B, the disintegrin-like, and the thrombospondin (TSP) type-1 domain with the final spacer. The central part shows the general relationship of ADAMTS proteins among vertebrates and is detailed in Figure 2. Each major monophyletic group has a bootstrap value of 1000, and are detailed in the adjacent ML trees. Species are color coded: pink for primates, red for non-primate placentals, orange for marsupials and monotremes, green for sauropsids, dark green for amphibians, purple for the coelacanth, dark blue for the gar with teleostan fish shown in blue and additionally in cyan for genes showing a 3R-WGD. Full phylogenies of the colored and peripheral genes are available in Additional file 1: Figure S1a to Figure S1h.

Phylogenetic analyses (Figure 2 and Additional file 1: Figure S1, and summary in Figure 3) showed that ADAMTS proteins are clustered into clades that match their structural subdivisions. These are: ADAMTS1, −4, −5, −8 and −15, which lack a PLAC domain and are known as the retrotransposed angiogenesis-aggrecanase group [14] (Additional file 1: Figure S1a1 and S1a2); ADAMTS9 and −20 (Additional file 1: Figure S1b); ADAMTS6 and −10 (Additional file 1: Figure S1c); ADAMTS16 and −18 (Additional file 1: Figure S1d); ADAMTS7 and −12 (Additional file 1: Figure S1e); ADAMTS17 and −19 (Additional file 1: Figure S1f); ADAMTS2, −3, and −14 (Additional file 1: Figure S1g), a group known as the procollagen amino propeptidases [14]; and ADAMTS13 (Additional file 1: Figure S1h). All of these groups were rooted by a single gene in either *Ciona* (*C. intestinalis* and/or *C. savignyi*) and/or amphioxus, or even from another branch of the Deuterostomia, the Echinodermata sea urchin *S. purpuratus* (Additional file 2: Figure S2 and Additional file 3: Figure S3). ADAMTS13 was found in a single copy in both Sarcopterygii and Actinopterygii species with these orthologous proteins clustered into an independent monophyletic group (Additional file 1: Figure S1h and see also Additional file 2: Figure S2 and Additional file 3: Figure S3). Thus, all genes except *ADAMTS13* remained duplicated at least once during the 2 successive WGDs, which occurred at the base of vertebrate evolution.

**Figure 4:**
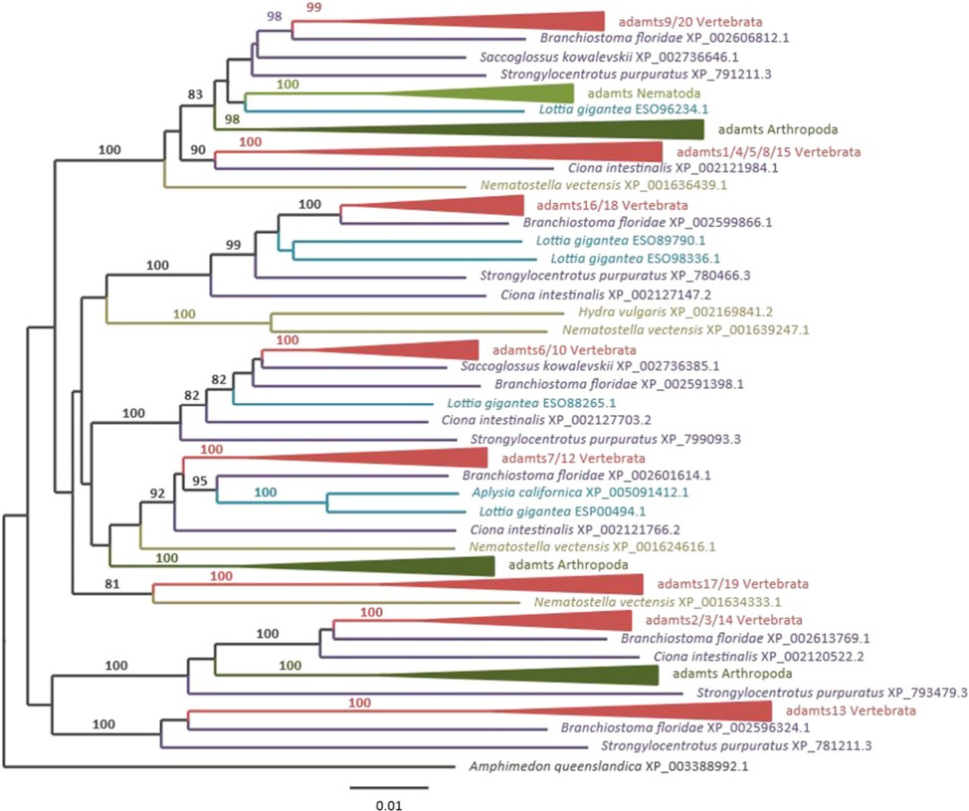
**Phylogenetic analysis of metazoan ADAMTS proteins.** Maximum likelihood (ML) values of full and best annotated sequences from metazoan species. Bootstrap replicates over 800 are shown. The full non-collapsed phylogeny is available in Additional file 2: Figure S2.

There were sets of two paralogous copies that were all rooted by one gene in non-vertebrate deuterostomian species: ADAMTS9 and −20, ADAMTS17 and −19, ADAMTS6 and −10, ADAMTS16 and −18, and ADAMTS7 and −12 (Figure 4 and Additional file 2: Figure S2 and Additional file 3: Figure S3). The dichotomic phylogenetic pattern they present is the signature of a duplication process that most likely occurred through either the 1R- or 2R-WGD, with no additional duplication in the teleost-specific 3R-WGD (Figures 2 and 3, and Additional file 1: Figure S1 and Additional file 2: Figure S2). Further, each of these pairs of paralogues share the common structure of all ADAMTS proteins; ADAMTS17 and ADAMTS19 have an additional four TSP type-1 domains ending with a PLAC domain, a structure also found in ADAMTS6 and its paralogue ADAMTS10. ADAMTS16 and ADAMTS18 have 5 and 4 TSP type-1 domains, respectively also ending with a PLAC domain. ADAMTS7 and ADAMTS12 have a similar structure with 3 TSP type-1 domains, a large spacer domain, four other TSP type-1 domains and a PLAC domain. ADAMTS9 and −20 are the two longest ADAMTS proteins with 14 additional TSP type-1 domains and a large GON domain to end the C-terminal region (Figure 1). Of note, the gene encoding *ADAMTS19* was lost along the lineage leading to Teleosteans, as it is not found in these fish, with the interesting caveat that it is observed in the gar genome (*Lepisosteus aculeatus*) (Additional file 1: Figure S1f).

**Figure 5:**
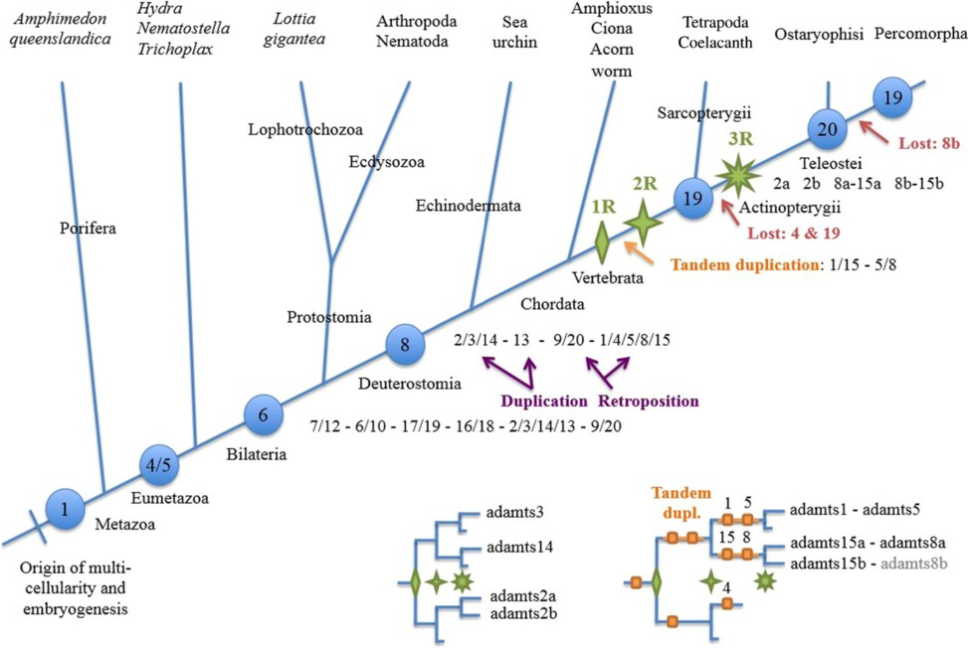
**Most parsimonious evolutionary history of the ADAMTS family.** This schematic representation proposes the expansion of the ADAMTS gene family starting from one sequence at the base of the Metazoa. Shown in purple is the duplication of the ancestral forms of adamts2/3/14 and adamts13, and the retroposition of the ancestral form of the genes adamts 9/20 into adamts1/4/5/8/15, which added from 6 to 8 the number of adamts genes from bilaterian to deuterostomian species. The 1R, 2R and 3R are shown by green stars with 2, 4 and 8 branches, respectively. The evolutionary history of the group of genes with teleostean duplicates is detailed. Shown in orange is the tandem duplication of the 1/5/15/8 gene while gene losses are shown in red.

The procollagen amino-propeptidases ADAMTS2, ADAMTS3 and ADAMTS14 also share a same common structure with three additional TSP type-1 domains ending by a PLAC domain (Figure 1). These three paralogues form a monophyletic group rooted together by orthologous proteins from non-vertebrate deuterostomes. In contrast to other family members, ADAMTS2 is found in two copies in each teleostean species, but only one copy in the non-teleost spotted gar, and also in the coelacanth and all tetrapod species (Additional file 1: Figure S1g and Additional file 2: Figure S2). The dichotomy found in the phylogeny is the pattern expected for the teleostean fish specific 3R-WGD. ADAMTS3 and ADAMTS14 are phylogenetically closer to each other than to ADAMTS2, indicating that their corresponding genes were most probably duplicated through the 1R- and 2R-WGD, with the counterpart of ADAMTS2 being lost after the second vertebrate WGD (Figure 5).

The last group of ADAMTS comprises ADAMTS1, ADAMTS4, ADAMTS5, ADAMTS8 and ADAMTS15. A single gene copy in the two urochordates roots all these vertebrate members (Figure 5 and Additional file 2: Figure S2 and Additional file 3: Figure S3). The phylogeny of these family members is complicated by an additional tandem duplication that has likely occurred early in the vertebrate evolution, most parsimoniously between the 1R- and 2R-WGD, as evident by *ADAMTS1* and *ADAMTS5* lying in a tail-to-head tandem configuration, and *ADAMTS8* and *ADAMTS15* lying in tail-to-tail tandem (Figure 5). ADAMTS5 and ADAMTS8 share the same structure with only one additional TSP type-1 domain ending the C-terminal region, while the two other members of the tandem duplications have 2 additional TSP type-1 domains. By contrast ADAMTS4 has no extra domain to end the C-terminal region after the spacer region (Figure 1). Thus, the ancestral (and unique) gene encoding this clade was duplicated during the 1R-WGD, one of the two copies went on to be duplicated in tandem before the occurrence of the 2R-WGD. In addition, it gained or lost one TSP-encoding sequence. The gene that lost its duplicate after that is *ADAMTS4*. As for the two tandem duplicates, one of the duplicates was inverted, ending in a different orientation (Figure 5). *ADAMTS4* was lost in the Actinopterygii lineage (in contrast to *ADAMTS19*, it is not even found in the spotted gar) but kept in the Sarcopterygii from the Coelacanth to all tetrapods (Additional file 1: Figure S1a1, S1a2, and Additional file 2: Figure S2). After the occurrence of the teleostean 3R-WGD, one of the copies of the tandem *ADAMTS1*-*ADAMTS5* was lost. The other tandem *ADAMTS8*-*ADAMTS15* remained duplicated in the lineage leading to the Ostariophysii (*adamts8a*-*adamts15a* and *adamts8b*-*adamts15b*) found in the zebrafish and the cave fish (*Astyanax mexicanus*), but *adamts8b* was lost in the lineage leading to the Percomorpha. Altogether in this clade, there are 5 *ADAMTS* in the Sarcopterygii (tetrapods), and among fish, 5 in Percomorpha and 6 in Ostariophysii.

Based on the alignment of the peptidase M12B, the disintegrin-like, the first TSP-type-1 and the cysteine-rich domains, phylogenetic analyses were performed and phylogenetic trees drawn at the same scale, which revealed some interesting features. A strong acceleration of the evolutionary pace of the ADAMTS20 proteins has occurred in the branch leading to the primates (Figure 3 and Additional file 1: Figure S1b and Additional file 2: Figure S2). A strong increase in the evolutionary pace is also observed in the branch leading to the therian for ADAMTS4 and ADAMTS14 (Figure 3, and Additional file 1: Figure S1a1, S1g, Additional file 2: Figure S2 and Additional file 3: Figure S3). ADAMTS2a and ADAMTS2b are evolving faster than their tetrapod counterparts, with a stronger acceleration of ADAMTS2a compared to ADAMTS2b in the Percomorpha lineage (Figure 3, and Additional file 1: Figure S1g, Additional file 2: Figure S2 and Additional file 3: Figure S3). ADAMTS6 is strongly constrained, with the opposite being true for ADAMTS13, which has the fastest evolutionary rate (Figure 3, and Additional file 1: Figure S1c, S1h, Additional file 2: Figure S2 and Additional file 3: Figure S3).

### Evolution of *ADAMTS* among Opisthokonta

The next aim of this study was to elucidate the evolutionary origin of all *ADAMTS* utilizing the data available in the non-vertebrate Opisthokonta, including insects or nematodes, that are ecdysozoan species, or lophotrochozoans (e.g. the mollusca *Lottia gigantea*), and even non-bilaterian species like the Cnidaria *Nematostella vectensis*. Only one sequence was retrieved in the Porifera, *i.e.* the sponge *Amphimedon queenslandica*, which was used to root the phylogenetic tree (Figure 4 detailed in Additional file 3: Figure S3). All but three groups of ADAMTS in vertebrates were in a monophyletic group shared with some *ADAMTS* genes found in either the Ecdysozoa or the Lophotrochozoa (Figure 4). The first exception was *ADAMTS13*, which encodes two CUB domains, a novelty probably acquired by domain shuffling at the base of the deuterostomian lineage from the procollagen amino-propeptidases-encoding ADAMTS2/3/14 (Figures 1 and 5). This scenario is consistent with an occurrence of a protein in the starlet sea anemone (XP_001628426.1) that can be annotated as ADAMTS2/3/14 (although this sequence is rather incomplete, data not shown). The second exception is the group of ADAMTS1/4/5/8/15 proteins, with a homologue found in *Ciona*, and a short sequence in the amphioxus (XP_002589343.1). Thus, this group of genes was acquired somewhere in the deuterostomian lineage leading to the Chordata (Figure 5). Given that ADAMTS9/20 belong to the same monophyletic group of both lophotrochozoans and ecdysozoans, the retroposition event most likely originated from the long *ADAMTS9*/*20*, a scenario more probable due to the fact that all of these genes are rooted by a Cnidaria sequence (Figure 4 and Additional file 2: Figure S2 and Additional file 3: Figure S3). The last group is *ADAMTS6*/*10*, which clusters with a very low bootstrap value with *ADAMTS7*/*12*, *ADAMTS16*/*18* and *ADAMTS17*/*19*. Therefore, a duplication of any of these in the bilaterian lineage could be a plausible evolutionary scenario (Figure 5).

### The *adamts* family is dynamically expressed throughout zebrafish embryonic development

**Figure 6:**
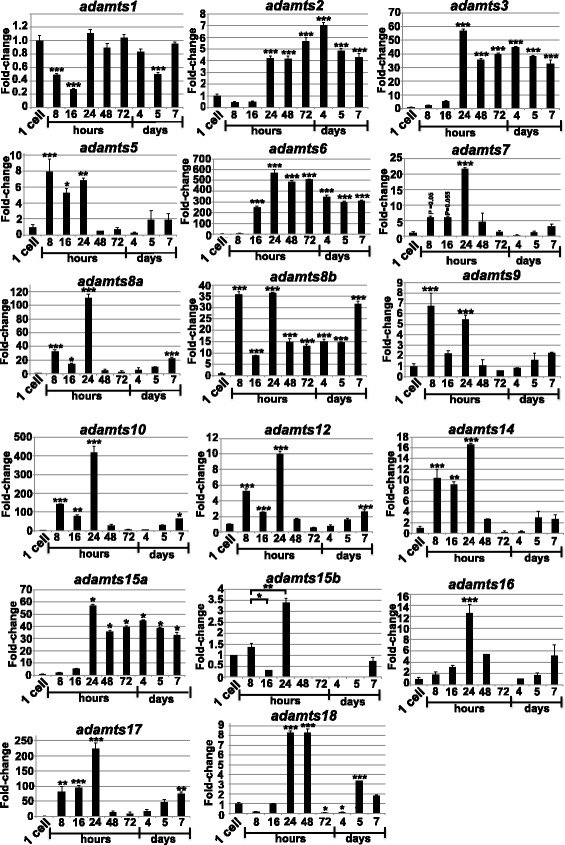
**Expression of adamts genes during zebrafish development.** Q-RT-PCR for each *adamts* gene from developmentally staged zebrafish embryo total RNA. Hours = hours post fertilization, Days = days post fertilization. The data are normalized to the 1 cell stage (zygote). *P < 0.05, **P < 0.01, ***P < 0.001.

Based on the detailed analysis of the evolutionary relationship of the ADAMTS family across vertebrate species, it became of interest to scope the potential of zebrafish as a model to study roles of ADAMTS enzymes in development. Therefore, Q-RT-PCR was performed on RNA extracted from staged zebrafish embryos to determine the expression profile of the *adamts* family, that revealed dynamic and overlapping expression patterns (Figure 6). Notably, with the exception of *adamts17,* all members of the family detected during embryogenesis were maternally derived with amplicons representing mRNA expression found at the one cell-stage. With the consideration of maternal mRNA instability in mind however, it is important to note that full-length maternally derived encoding mRNA transcripts for each *adamts* gene detected were not confirmed at this stage. In most cases *adamts* mRNA species were however upregulated at 8 hours post fertilization (hpf) indicative of zygotic expression with *adamts5, adamts7, adamts9* and *adamts12* increasing 5-fold or greater; *adamts8a, adamts8b, adamts14, adamts17* increasing 10-fold or greater, and; *adamts6* and *adamts10* increasing 100-fold or greater (Figure 6). During somitogenesis (16 hpf) noticeable increases in mRNA expression were observed for *adamts3*, *adamts6*, *adamts15a*, *adamts16* and *adamts18* (Figure 6). All *adamts* members detected showed significantly increased mRNA expression at 24 hpf coinciding with the onset of organogenesis (Figure 6). In contrast, mRNA transcripts for *adamts13* were not detected despite attempts with several sets of DNA oligonucleotide primers and varying RT-PCR conditions (data not shown)*.* However, there is strong bioinformatics evidence of its presence in the zebrafish genome (Figure 2 and Additional file 1: Figure S1, Additional file 2: Figure S2 and Additional file 3: Figure S3).

### *Adamts15a* and *adamts9* are specifically expressed in craniofacial and neural structures respectively during zebrafish embryonic development

**Figure 7:**
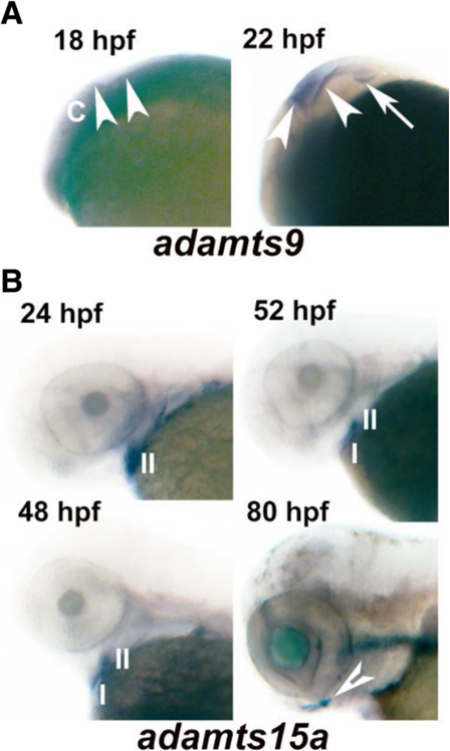
**Expression of adamts9 and adamts15a genes during zebrafish development.** Whole-mount *in situ* hybridisation of *adamts9* and *adamts15a* in developing zebrafish embryos. **A**. *adamts9* is expressed in the cerebellum (18 and 22 hpf - arrow heads) and rhombic lip (22 hpf - arrow). **B**. *adamts15a* is expressed in branchial arch II (24 hpf), branchial arch I and II (48 and 52 hpf) and Meckel’s cartilage (80 hpf - open arrow). Expression was confirmed using two separate mRNA probes to each gene. hpf = hours post fertilization.

There is currently no report of an *Adamts15* knockout mouse, and the *Adamts9* knockout mouse is embryonic lethal at gastrulation, making the zebrafish a prime candidate to study their roles in development. Therefore, whole-mount *in situ* hybridisation was performed for *adamts15a* and *adamts9* to obtain a snapshot of the expression pattern of two key members of the proteoglycanase clade of ADAMTS enzymes. *adamts9* was specifically expressed in neuronal tissue at 18 hpf and 22 hpf, in the cerebellum and rhombic lip respectively (Figure 7A), and was also found in the ventral region of the otic vesicle at 22 hpf (Figure 7A – right hand panel). For *adamts15a*, expression could be seen in the hyoid (2nd arch) at 24 hpf with additional expression in the 1st arch (Meckels cartilage) from 48 hpf through to 80 hpf (Figure 7B).

### The *adamts* family is widely expressed in zebrafish adult organs

**Figure 8:**
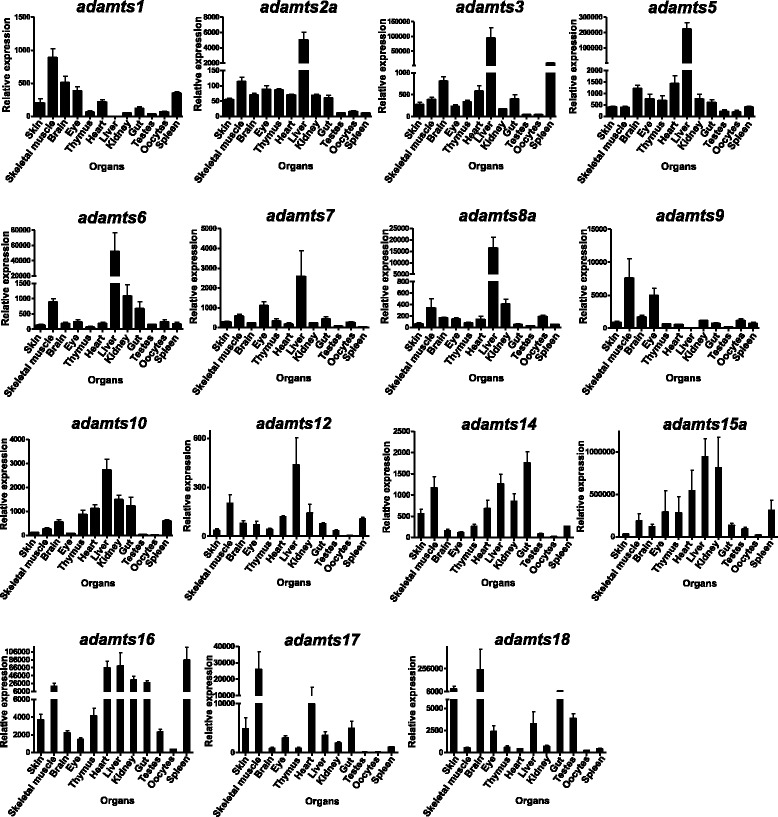
**Expression of adamts genes in adult zebrafish organs.** Q-RT-PCR for each *adamts* gene from zebrafish adult organ total RNA. The data are presented as relative levels. One way ANOVA and Tukey’s post-hoc analyses are available in Additional file 5.

Further analysis of *adamts* expression in adult zebrafish by RT-PCR revealed a wide distribution across different organs (Additional file 4: Figure S4) suggesting a role for those genes in normal adult organ function. Subsequent Q-RT-PCR performed across organs revealed some interesting expression patterns. High levels of expression were seen in the liver for *adamts2a*, *adamts3*, *adamts5*, *adamts6*, *adamts8a*, *adamts10*, *adamts15a*, *adamts16* and *adamts18* (Figure 8). *adamts1* and *adamts9* were most highly expressed in skeletal muscle but both essentially absent in the liver. *adamts9* was also highly expressed in the eye (Figure 8). The brain had representative transcripts for most *adamts* genes with notably high expression in the case of *adamts18* (Figure 8). *adamts15a* was the most highly expressed gene in several organs including skeletal muscle, eye, thymus, heart, liver, kidney and gut (Figure 8).

## Discussion

This report describes a significantly updated evolutionary understanding of ADAMTS metzincins across vertebrate species compared with previous studies [12-14]. The initial motivation of this work was to check whether each of these genes had an orthologue in the zebrafish and/or the medaka, two fish species that represent two major species groups that diverged about 130 millions of years ago (MYA) and circa 65 MYA, respectively [21]. Given that the vertebrate lineage experienced two WGDs at its base [18,19] and a third WGD specific to the fish teleost species [17,20], it was of interest to determine how many ohnologues (paralogues originating from a WGD) could be found in these fish compared to human, and, whether the evolutionary scenario of the ADAMTS in the metazoans could be elucidated. The *ADAMTS* family, composed of 19 genes in the human and mouse, has expanded dramatically during the course of Metazoa evolution starting from one single gene in the common ancestor with the sponge, more than 600 MYA [22], up to 8 in the non-vertebrate chordates (Figure 6). Future releases of genomes in species may lead to slight modifications in the proposed scenario at deeper animal nodes; however, the conservative approach used here should minimize them.

The 1R-and 2R-WGDs have substantially impacted this gene family in the vertebrate lineage expanding its numbers to 19. Interestingly, the teleost specific 3R-WGD did not have a significant impact on the number of *ADAMTS* genes as the duplicates retained (*ADAMTS2* and *ADAMTS15* in teleosts, and *ADAMTS8* in the Ostariophysii) may be compensating the general teleostean losses of *ADAMTS4* and *ADAMTS19*, albeit that *ADAMTS19* is still found in the gar.

In general, phylogenetic trees demonstrated very similar patterns of branching and branch lengths across *ADAMTS* genes. The first exceptions are teleost *ADAMTS2a* and *ADAMTS2b* that both have very long branches compared to the tetrapod orthologue *ADAMTS2* indicating the former two are rapidly evolving possibly gaining divergent functions, a pattern of sub-functionalization. The second is *ADAMTS20*, which has a very distinctive branch in the primates, suggesting specific divergence.

We previously reported the remarkable conservation of certain ADAMTS members (ADAMTS5, Genbank JF778846.1; ADAMTS9, Genbank JF778848.1; ADAMTS15a, JF778847.1) at the primary amino acid level [23]. Ancestral *ADAMTS* gene function has been previously reported in early evolution with *C. elegans gon-1* shown to mediate distal tip migration during gonadal morphogenesis [6]. Moreover, ADAMTS knockout mice show profound developmental defects demonstrating their unquestionable importance during vertebrate embryonic development.

ADAMTS1 knockout mice have reduced rates of ovulation and impaired fertility as well as abnormally remodelled ovarian follicular basement membranes [24-26]. We found a low abundance of *adamts1* transcript in oocytes but did not investigate its abundance in follicular somatic cells in this current study, although its presence has been previously reported in teleost fish follicular somatic cells [27]. Therefore it may have similar roles in the ovulatory process across vertebrate species.

During limb development, reduced interdigital tissue resorption leading to soft tissue syndactyly (webbed feet) occurred in combinatorial ADAMTS5, ADAMTS9 and ADAMTS20 knockout mice and mesodermal *Prx-1* Cre conditionally deleted ADAMTS9 (which is non-redundant in this developmental context) [28,29]. In this current study, *adamts1*, *adamts5* and *adamts15* were abundant in adult zebrafish skeletal muscle and we previously reported a role for ADAMTS5 and ADAMTS15 during myogenesis and muscle regeneration [30].

Cardiac and aortic abnormalities including the appearance of ectopic chondrogenic nodules and myxomatous heart valves were observed in heterozygous (*Adamts9* +/−) mice [31] and myxomatous heart valves were also observed in *Adamts5−/−* mice. Both *Adamts5* and *Adamts9* were expressed in the adult mice hearts, although whole-mount *in situ* hybridisation for *adamts9* did not reveal clearly discernable expression in the zebrafish heart at the developmental time-points examined in this study. However, in adult zebrafish hearts, *adamts5* and *adamts15a* were strongly expressed suggesting a role for these two genes in normal heart function.

*Adamts9*−/− (homozygous) knockout mice were embryonic lethal, dying around gastrulation [32] and its expression was found in mesoderm near the optic vesicle and in the common ventricle in the mouse [33]. This current study showed strong expression of *adamts9* in the zebrafish adult eye and developing otic vesicle, and consistent with the mouse, in the primitive central nervous system including the rhombic lip and presumptive cerebellum.

Interestingly, single ADAMTS4 knockout mice have no apparent phenotype although when knocked out combinatorially with *Adamts1*, mice die of post-natal renal failure [34]; the current study shows *adamts4* is absent in the zebrafish genome, collectively suggesting ADAMTS4 to be less important during vertebrate development, whereas *adamts1* was strongly expressed in zebrafish kidney. Several *adamts* genes were highly expressed in the adult zebrafish liver, however roles for ADAMTS enzymes in liver function are largely unreported with the exception of a report of their expression correlating with liver fibrosis [35].

We recently reported the expression of *Adamts15* during mouse embryogenesis [36]; however, there is currently no report of an *Adamts15* knockout mouse. In this current study, we showed specific expression of *adamts15a* in the first and second pharyngeal arches of the zebrafish, suggesting a role during craniofacial development. While little is known about *Adamts15* in mouse craniofacial development, *Adamts5* is expressed in Meckel’s cartilage in the developing mouse embryo [37], while *Adamts9* is expressed in branchial arches I and II in the mouse [33]. Each of these enzymes have overlapping substrate specificity towards versican, a widely expressed transitional matrix proteoglycan [8]. Therefore it is attractive to hypothesise, given the cooperative nature of the ADAMTS family that more prominent roles for any given family member could arise through divergent evolution.

In each of the ADAMTS proteoglycanase (the retro-transposed aggrecanases and ADAMTS9 and ADAMTS20) knockout mice outlined above versican remodelling is markedly reduced or essentially absent in areas relevant to their phenotypes. The zebrafish genome possesses two confirmed versican homologues: versican (*vcanb*) and dermacan (*dcan*) [38] and one computationally predicted versican homologue (*vcana*). The putative zebrafish versicans (*vcana* and *vcanb*) both represent only a small portion of mammalian versican, comprising just a G3 globular domain. However, this domain is known to mediate important cellular events such as proliferation, and both resistance and sensitivity to apoptosis during tumourigenesis [39,40]. Dermacan, on the other hand, shows homology to all regions of full-length human versican (V0 splice variant) including the N-terminal region proteolytically processed by mammalian ADAMTS proteoglycanases (data not shown). Although the specific ADAMTS–mediated versican cleavage site reported: DPEAAE↓A [41] is not conserved in the zebrafish, an alternatively conserved cleavage site: VAEQE↓A is present. Whether ADAMTS-mediated remodelling of dermacan is apparent in the zebrafish throughout development requires further investigation, although dermacan expression overlaps with *adamts15* expression in zebrafish craniofacial development [38].

## Conclusions

Our data highlight the evolution of the *ADAMTS* gene family through recurrent events of single gene duplication processes across metazoans with by a burst of amplification through vertebrate WGD events. In contrast, the WGD event that occurred at the base of the teleostean fish did not impact much the total number of this gene family in fish compared to other vertebrates. ADAMTS enzymes are highly conserved across vertebrate species, dynamically expressed during zebrafish embryogenesis and widely expressed in its adult organs. Given the incontrovertible role of some ADAMTS enzymes, and in particular the proteoglycanases during murine embryonic development, it is likely that similar and additional roles exist in the developing zebrafish embryo. The zebrafish therefore represents an additional powerful model to further our knowledge regarding the underlying complexities of ADAMTS enzyme biology in development and disease. It is also expected to help in understanding the increasing complexity and specialisation of functions of these enzymes during evolution since our common ancestor with sponges.

## Methods

### Bioinformatics

Protein sequences of ADAMTS family members across Chordata were retrieved from 3 databases (DB): Ensembl (v70), Uniprot (SwissProt + TrEMBL) and nr-prot (NCBI). Sequences from representative species were aligned using MAFFT [42]. Using *ad hoc* scripts and manual selection, multiple sequences in each species were trimmed, retaining the best-annotated sequences in those alignments. To root the phylogenies, when available and pending their ability to be aligned, the sequences from *Ciona intestinalis*, *Ciona savignyi*, *Branchiostoma floridae*, and from outside Phylum Chordata (e.g. *Strongylocentrotus purpuratus*, *Saccoglossus kowaleskii*, *Crassostrea gigas* or insects) were used. Phylogenetic analyses were achieved using the methods of Neighbor-Joining (BioNJ) [43] implemented in SeaView [44], or run under a WAG model with estimated gamma distribution parameter for the Maximum Likelihood (PhyML) [45], both of them with 1000 bootstrap iterations. In addition to our phylogenetic analyses, the orthologous relationship to each of these genes in the vertebrates was verified using Genomicus [45,46], which allows the visualization of orthology by synteny from the genomic information provided by the Ensembl database. BioNJ values (available upon request) corroborated those obtained by PhyML but were not added to keep clarity in the figures.

We also looked for adamts proteins beyond Chordata and to discriminate them from other metalloproteinases such as those encoding ADAM genes, common protein sequences of each ADAMTS encompassing the metalloproteinase, the disintegrin-like, the first TSP type-1 and the cysteine-rich domains were selected. Each of these sequences was used in BLASTP (blosum45 matrix used) searching of the nr protein database at NCBI restricted to the non-vertebrate Opisthokonta species. Retrieved protein sequences were pooled together with the vertebrate ADAMTS sequences, with sequences retained that aligned best all along these vertebrate sequences. This very stringent selection process was important to understand the relationship of all ADAMTS in species very distantly related and with different modes of development, and obtain an overview of the whole evolutionary history; the caveat being that it may have discarded ADAMTS genes that followed a different evolutionary path in other species, such as the ecdysozoans and the lophotrochozoans.

### Ethics

All studies on zebrafish (*Danio rerio*) were carried out with the approval of the Animal Welfare Committee, Deakin University in accordance with the National Health and Medical Research Council, Australia guidelines for care and use of animals in research.

### Zebrafish embryo and tissue collection and ribonucleic acid (RNA) extraction

Laying beds were placed into harem tanks containing one male per two females the night before embryo collection. Embryos were collected in Petri dishes following light-induced spawning and sorted to remove non-viable offspring, then routinely transferred into 0.003% w/v 6-n-propyl thio-uracil (PTU) (Sigma-Aldrich, Australia) in system water and incubated at 28°C. Time-staged embryos were humanely sacrificed by adding 10% Benzocaine (Sigma-Aldrich) drop-wise to Petri dishes containing embryos, after which the embryos were transferred to Trizol (Life Technologies, Australia) for RNA extraction or fixed in 4% paraformaldehyde (PFA) for *in situ* hybridization. Alternatively, specific organs and tissues were dissected from humanely sacrificed adult zebrafish and transferred to Trizol.

### Quantitative Reverse Transcription Polymerase Chain Reaction (Q-RT-PCR) amplification of zebrafish mRNA

RNA was extracted from Trizol samples as per manufacturer’s recommendations and stored at −80°C until further processing. Total RNA concentrations were obtained using a NanoVue spectrophotometer (GE Healthcare, Buckinghamshire, UK) with an A_260_/A_280_ nm ratio of ≥1.8 deemed acceptable for downstream analysis. Total RNA was reverse-transcribed into cDNA using a Superscript III (Life Technologies) or iScript cDNA synthesis kit (Bio-Rad, Australia) using random hexamers. RT-PCR was performed using *Taq* DNA polymerase (Roche) to ensure the specificity of the RT-PCR products before performing Quantitative RT-PCR (Q-RT-PCR) (Additional file 3: Figure S3). For sequencing, RT-PCR products were subjected to electrophoresis on 1.5% agarose gels in 1X TAE buffer and bands were excised and gel purified using a Gel Purification Kit (Life Technologies) for ligation into pGEMTeasy (Promega, Australia). Positive clones were sequenced using vector specific (T7 and SP6) primers at the Australian Genome Research Facility (AGRF) (Walter and Eliza Hall Institute of Medical Research, Melbourne, Australia). Q-RT-PCR was performed on the cDNA using iQ SYBR Green Supermix (Bio-Rad) and respective primers for the genes of interest (sequences available upon request) as per the manufacturer’s recommendations. Due to potential instability of mRNAs expressed during early development, input cDNA levels were normalised using the Oligreen assay (Life Technologies) as per previous studies that have examined gene expression across embryonic development and CT values were then normalised to input cDNA levels. The deltaCT method was used to compare changes in mRNA transcript levels across developmental time-points [30,47]. DeltaCT values for each gene was then normalised to their respective levels at the 1-cell embryonic stage. For Q-RT-PCR of adult organs, deltaCT levels were normalised to input cDNA levels to generate arbitrary units that were presented across organs to give an indication of relative changes in gene expression.

### Whole-mount *in situ* hybridisation

Whole-mount *in situ* hybridization was performed as previously described [48,49]. Linearized pGEMTeasy templates containing *adamts9* or *adamts15a* cDNA inserts amplified as described above were column purified and 1 μg of template used for *in vitro* transcription of digoxygenin-labelled sense or antisense RNA probes. Embryos were imaged on an Olympus, MVX10 dissecting microscope.

### Statistics

Q-RT-PCR results were analysed by one-way ANOVA with Tukey’s *post hoc* analysis using the SPSS or GraphPad statistical software packages. P < 0.05 was considered statistically significant. P values for comparative levels of gene expression across adult organs are available in Additional file 5.

### Availability of supporting data

The data sets supporting the results of this article are available in the Dryad repository, http://datadryad.org/review?doi=doi:10.5061/dryad.57sg5 [50].

## Additional files

Additional file 1: Figure S1. Phylogenetic analysis of proteins of the adamts family in vertebrate species. Phylogenetic analysis by maximum likelihood (ML) of all genes of the adamts family based on the shared structure being the metallopeptidase M12B, the disintegrin-like, the thrombospondin (TSP) type-1 domain with the final spacer. 1a1 and 1a2: ADAMTS1, −4, −5, −8 and −15. 1b: ADAMTS9 and −20. 1c: ADAMTS6 and −10. 1d: ADAMTS16 and −18. 1e: ADAMTS7 and −12. 1f: ADAMTS17 and −19. 1 g: ADAMTS2, −3, and −14. 1 h: ADAMTS13. Species are color coded: pink for primates, red for non-primate placentals, orange for marsupials and monotremes, green for sauropsids, dark green for amphibians, purple for the coelacanth, dark blue for the gar with teleostan fish shown in blue and additionally in cyan for genes showing a 3R-WGD. The leaflet versions of these trees are used in the peripheral ML trees in Figure 3. Additional file 2: Figure S2. Phylogenetic analysis of proteins of the adamts family in vertebrate species. Phylogenetic analyses by maximum likelihood (ML) of all genes of the adamts family based on the largest shared protein sequences. Sarcopterygii are colored in red, Actinopterygii and in blue, two blue colors are used for 3R-WGD duplicated genes in teleosts, non vertebrate species are in green. Protein IDs are attached to the gene name and organism species. Additional file 3: Figure S3. Phylogenetic analysis of proteins of the adamts family in metazoan species*.* Only full and best aligned sequenced were analyzed using maximum likelihood (ML). Branch values show the bootstrap replicates. This large phylogeny is available as a Newick formatted tree that can be opened with any software displaying phylogenetic tree (e.g. Seaview [41]). The collapsed version of this Newick format tree is shown in Figure 4. Additional file 4: Figure S4 Expression of adamts genes in adult zebrafish. RT-PCR of ADAMTS enzymes in adult zebrafish tissues. Total RNA was isolated from adult zebrafish organs and RT-PCR was performed for each *adamts* enzyme with same primer sets used for quantitative RT-PCR. β- actin was used as a house-keeping gene. Additional file 5: Figure S5 Statistical analyses of Q-RT-PCR results obtained for adult zebrafish organs. Data obtained by Q-RT-PCR for each *adamts* gene was analyzed by One-way ANOVA with Tukey’s post-hoc analyses using GraphPad statistical software. Resultant P-value tables were exported into Microsoft Excel spreadsheets. Each tab represents an individual *adamts* gene with an identifying legend for zebrafish organs.
