# Supplementary figures and images for: The evolutionary conservation of the A Disintegrin-like and Metalloproteinase domain with Thrombospondin-1 motif metzincins across vertebrate species and their expression in teleost zebrafish
Source: BMC Evol Biol. 2015 Feb 15;15:22. doi: 10.1186/s12862-015-0281-9 (PMC4349717; doi:10.1186/s12862-015-0281-9)

1a1

adamts1

947

adamts4

adamts8

adamts15  
adamts5

0.06

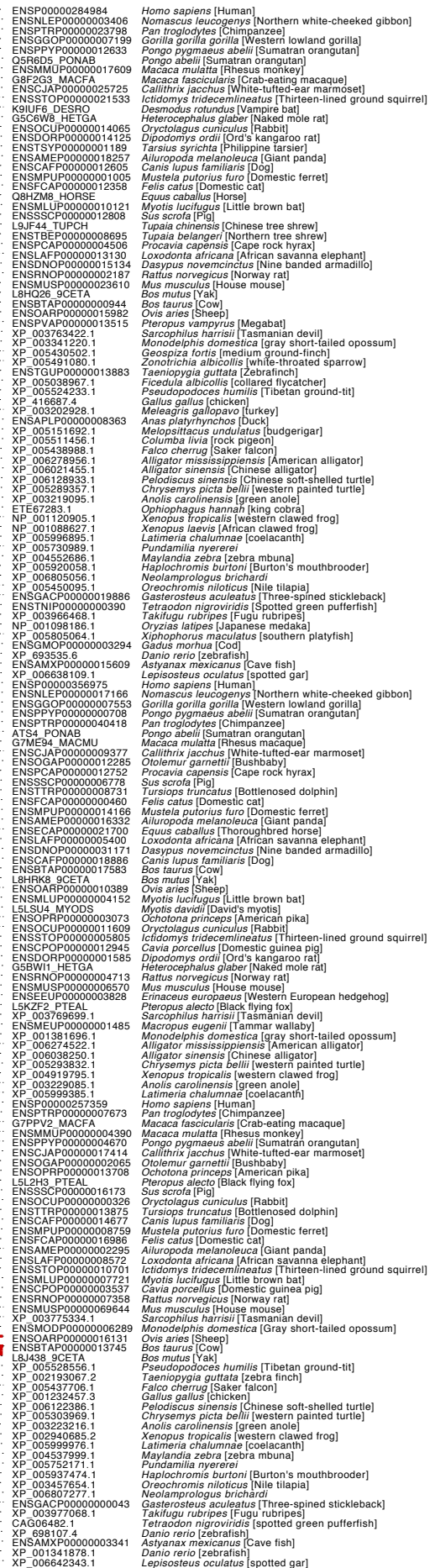

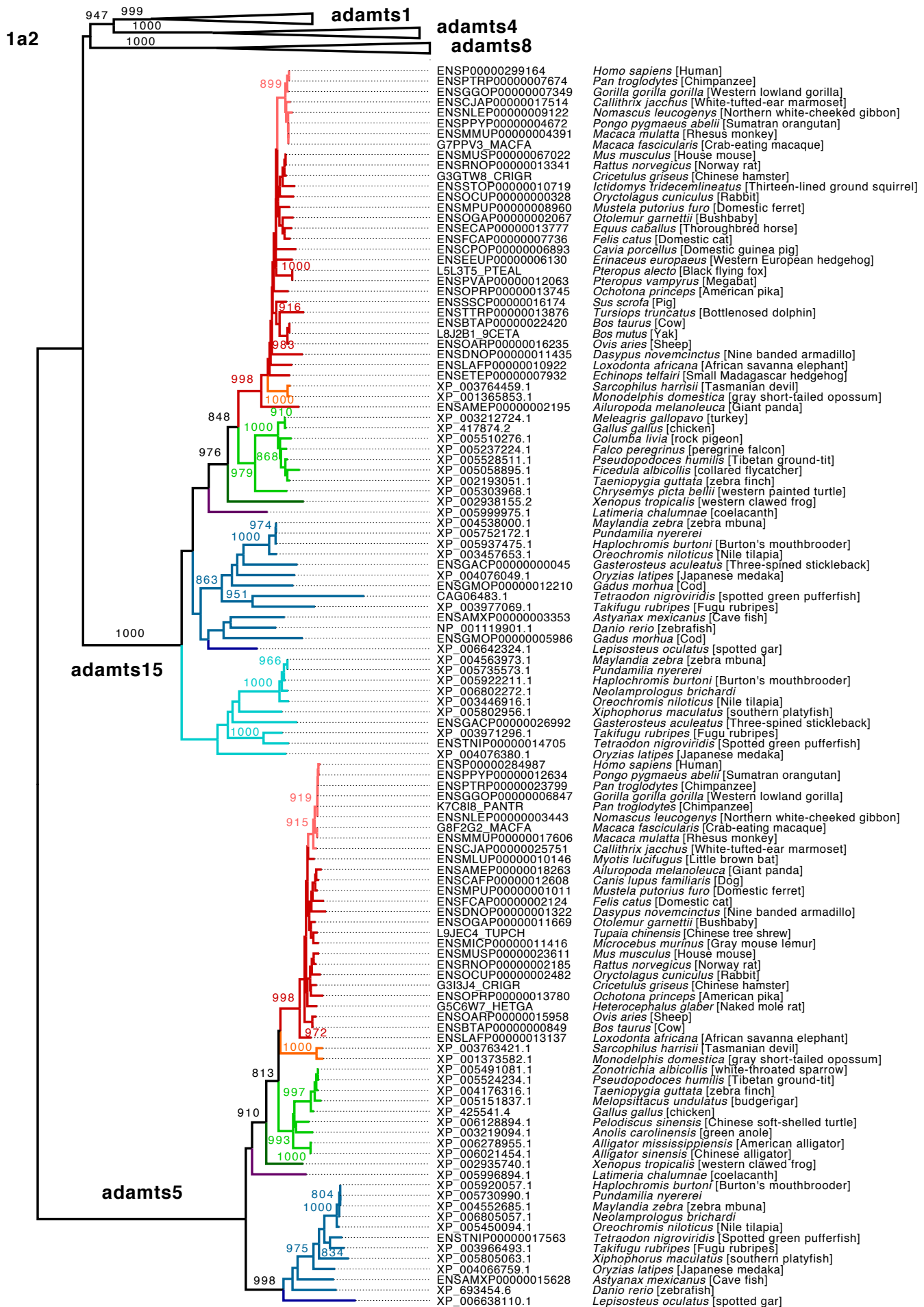

1b

adamts9

adamts20

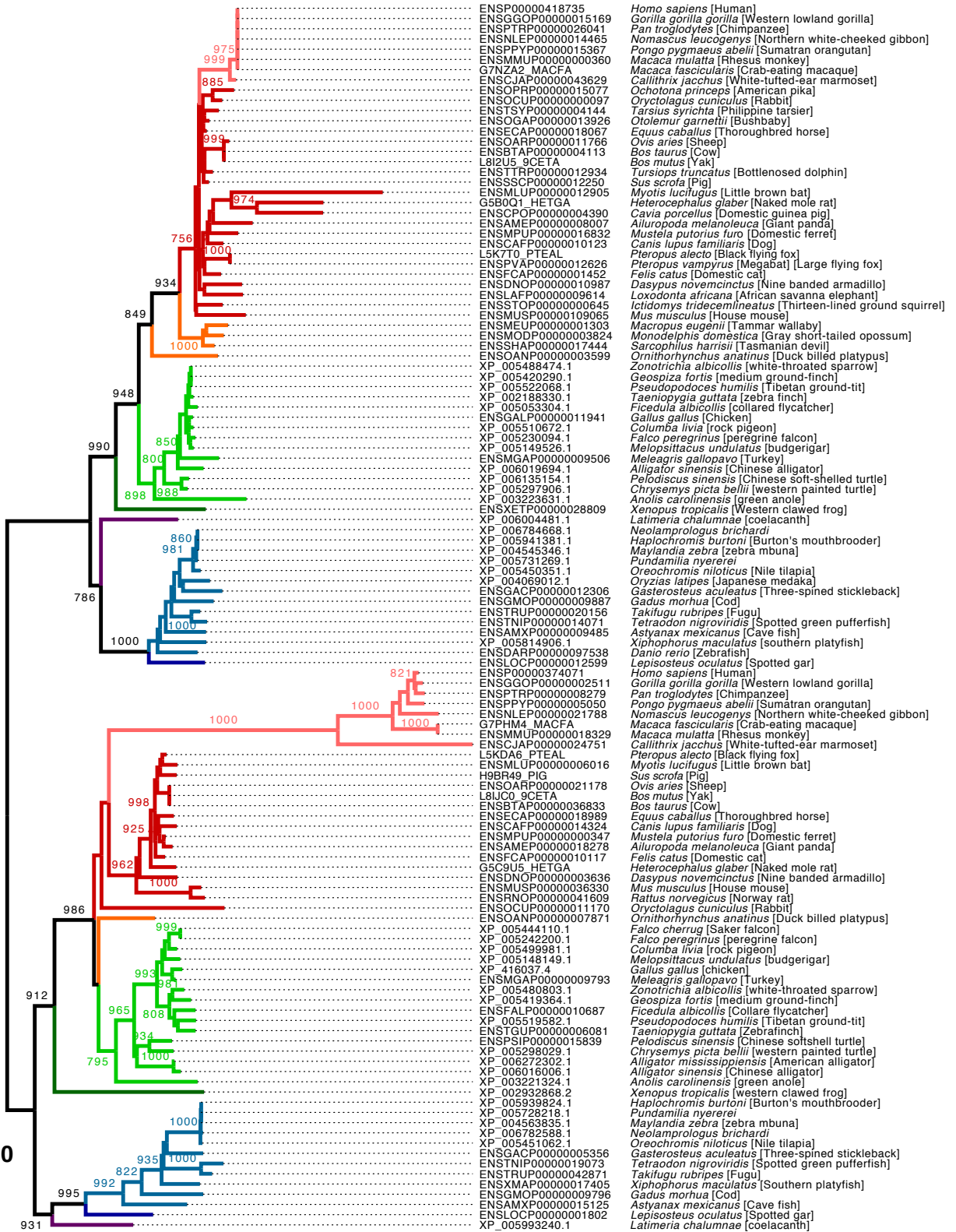

0.04

**1c**

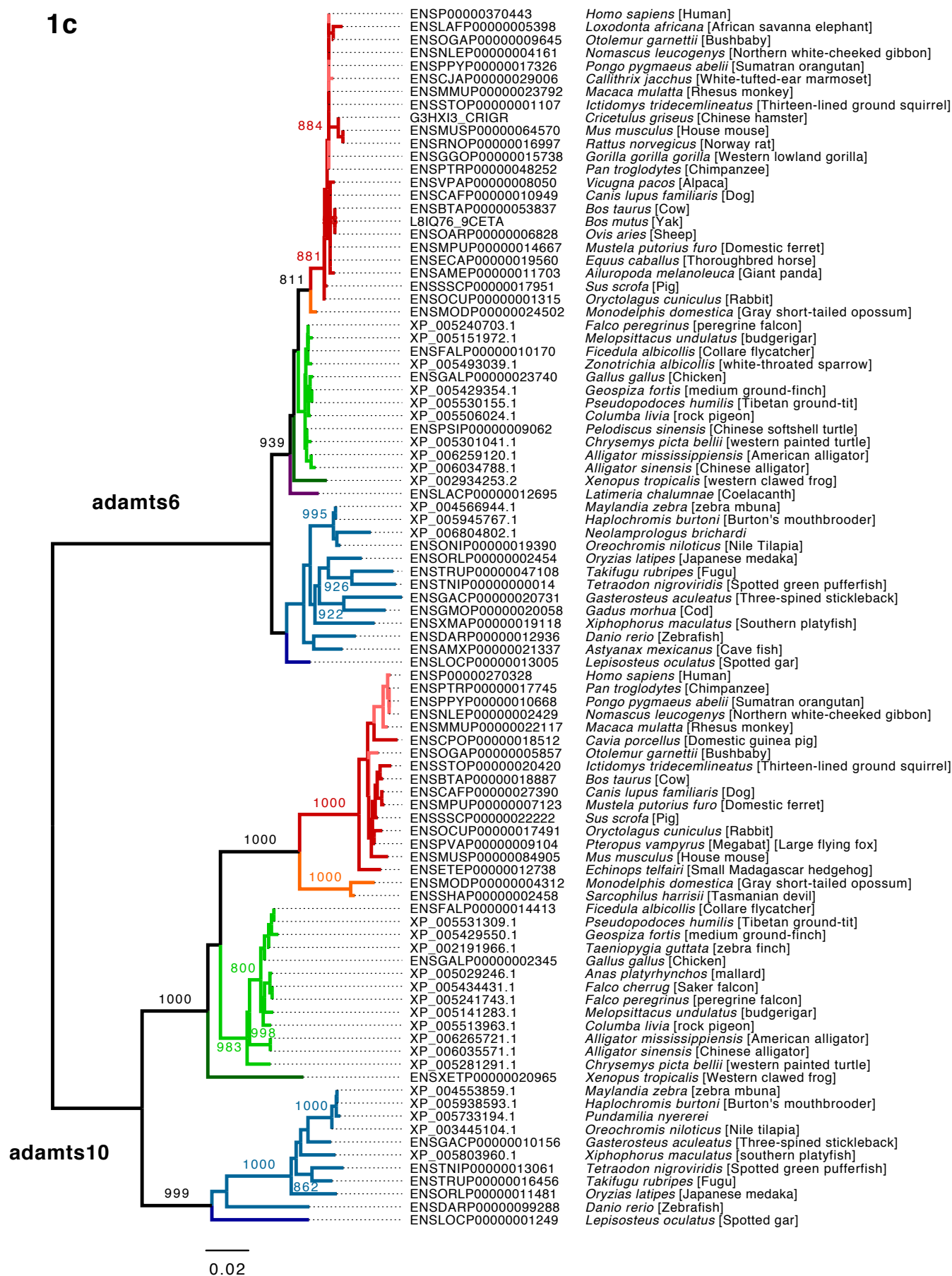

1d

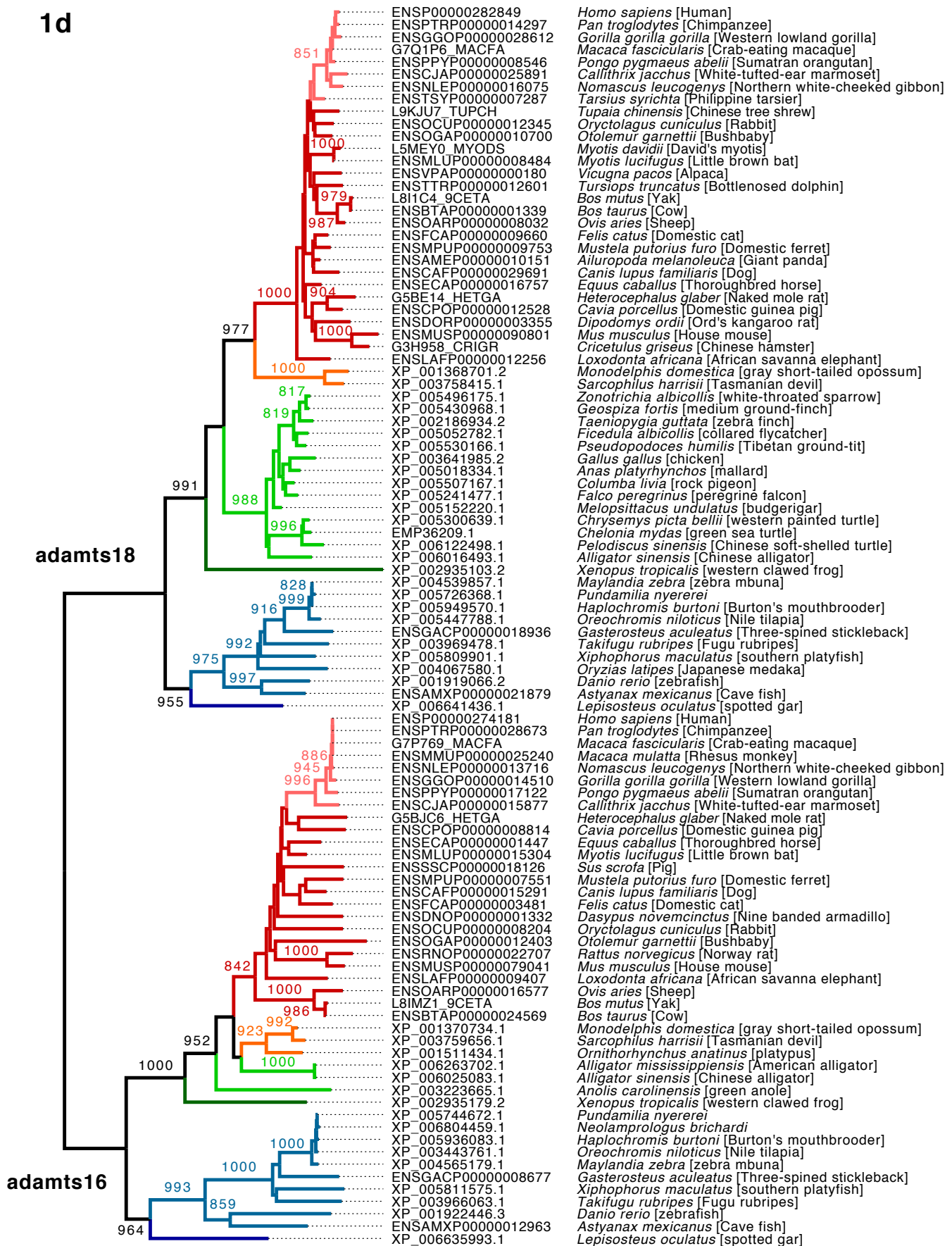

0.03

1e

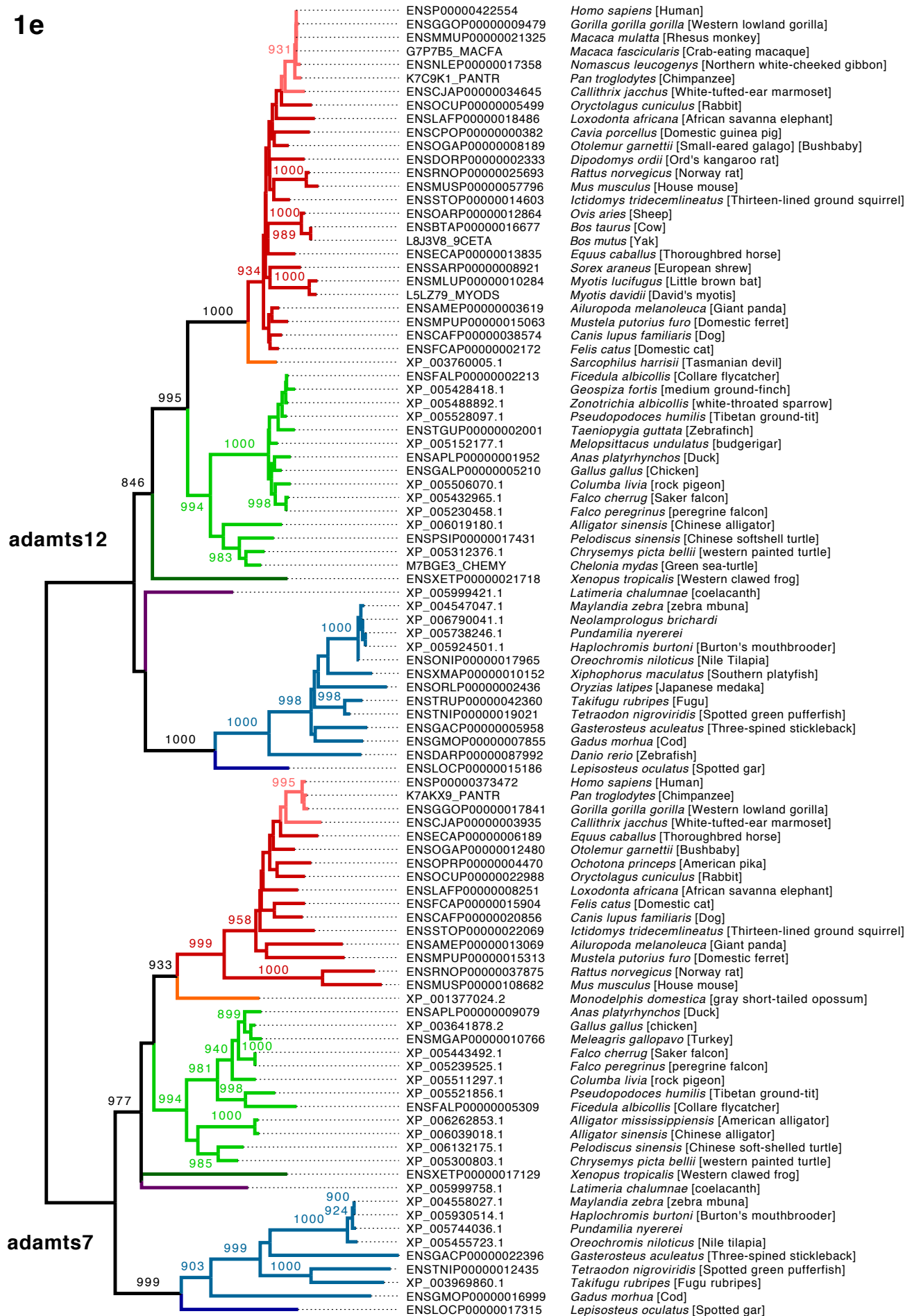

1f

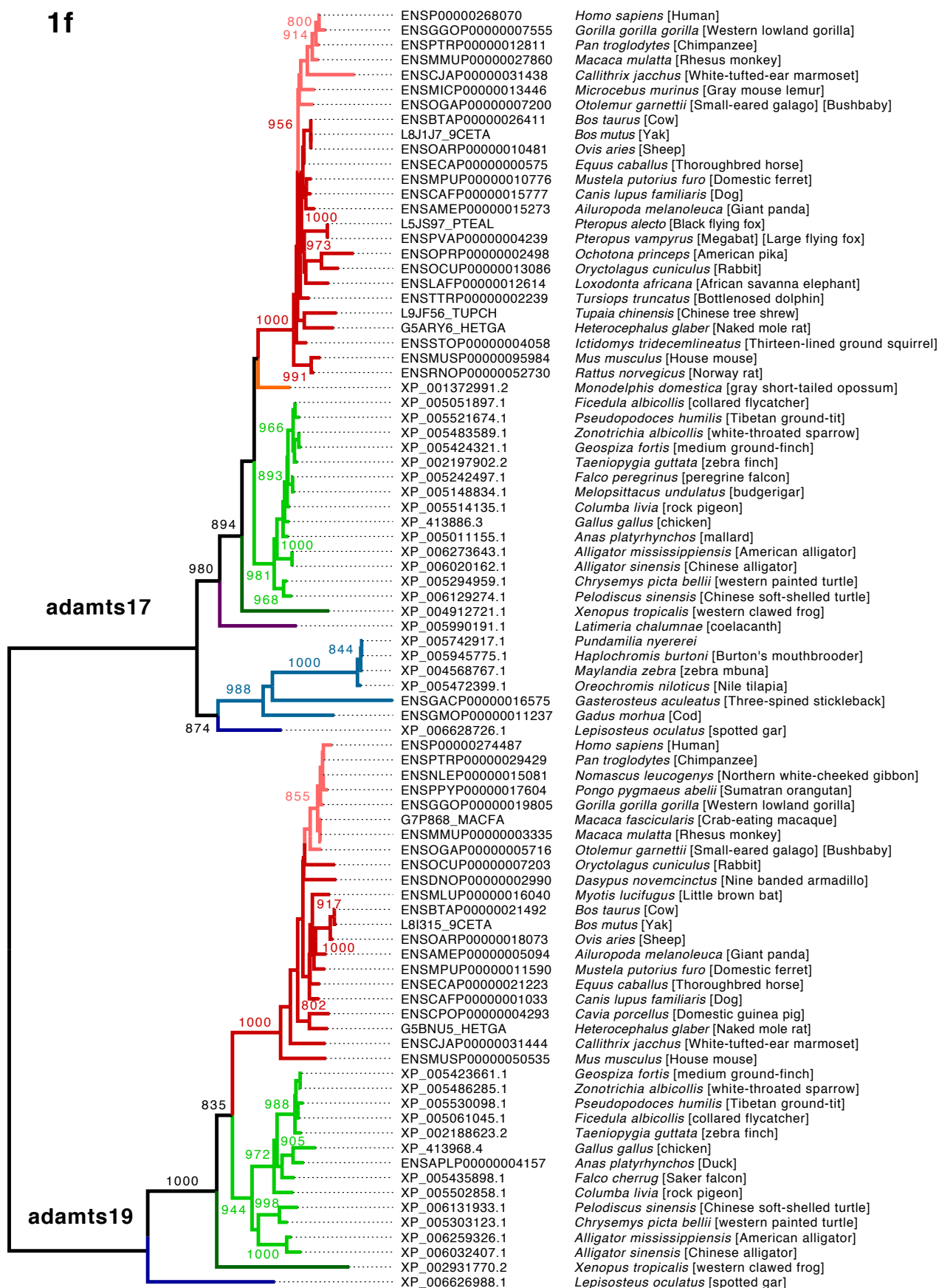

0.04

**1 g**

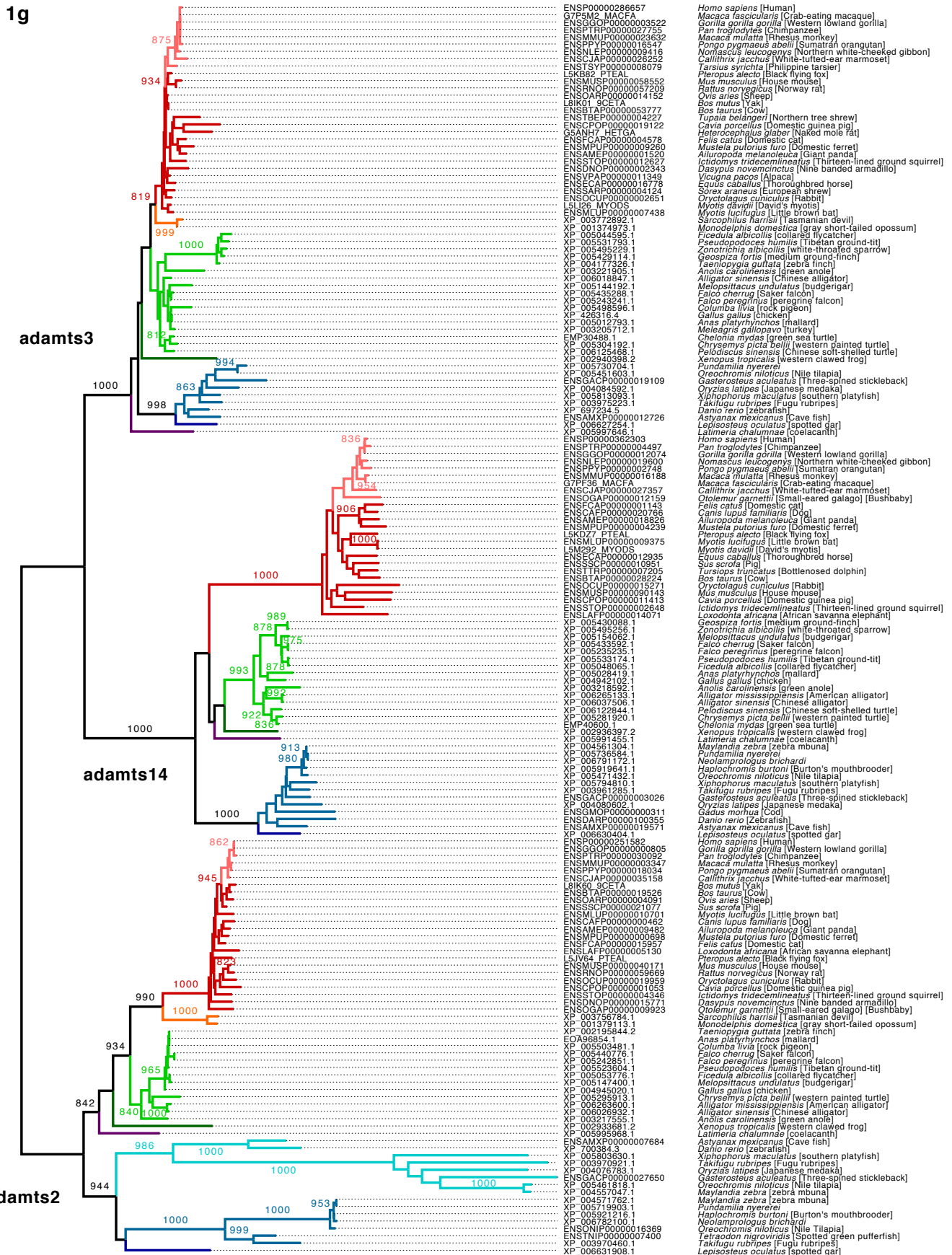

0.04

1h

adamts13

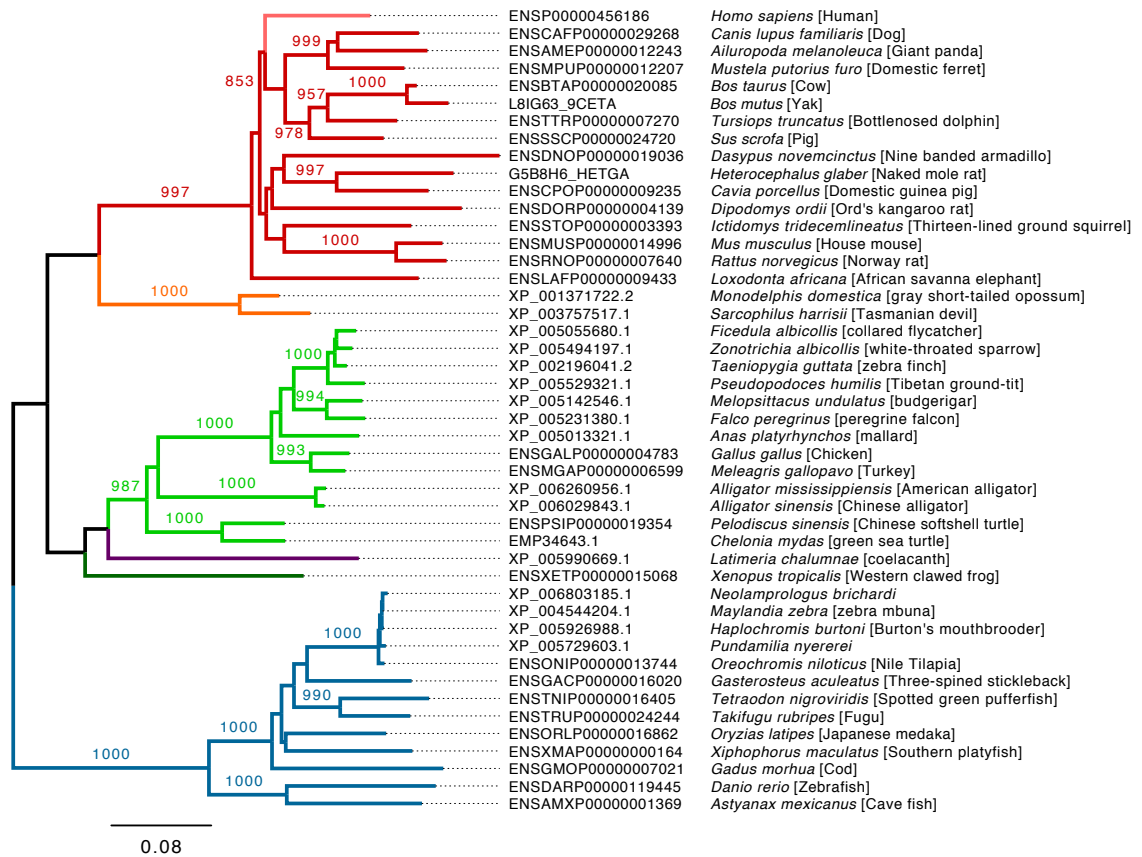

Supplement: Additional file 1: Figure S1. — Phylogenetic analysis of proteins of the adamts family in vertebrate species. Phylogenetic analysis by maximum likelihood (ML) of all genes of the adamts family based on the shared structure being the metallopeptidase M12B, the disintegrin-like, the thrombospondin (TSP) type-1 domain with the final spacer. 1a1 and 1a2: ADAMTS1, −4, −5, −8 and −15. 1b: ADAMTS9 and −20. 1c: ADAMTS6 and −10. 1d: ADAMTS16 and −18. 1e: ADAMTS7 and −12. 1f: ADAMTS17 and −19. 1 g: ADAMTS2, −3, and −14. 1 h: ADAMTS13. Species are color coded: pink for primates, red for non-primate placentals, orange for marsupials and monotremes, green for sauropsids, dark green for amphibians, purple for the coelacanth, dark blue for the gar with teleostan fish shown in blue and additionally in cyan for genes showing a 3R-WGD. The leaflet versions of these trees are used in the peripheral ML trees in Figure 3. [file 12862_2015_281_MOESM1_ESM.pdf]

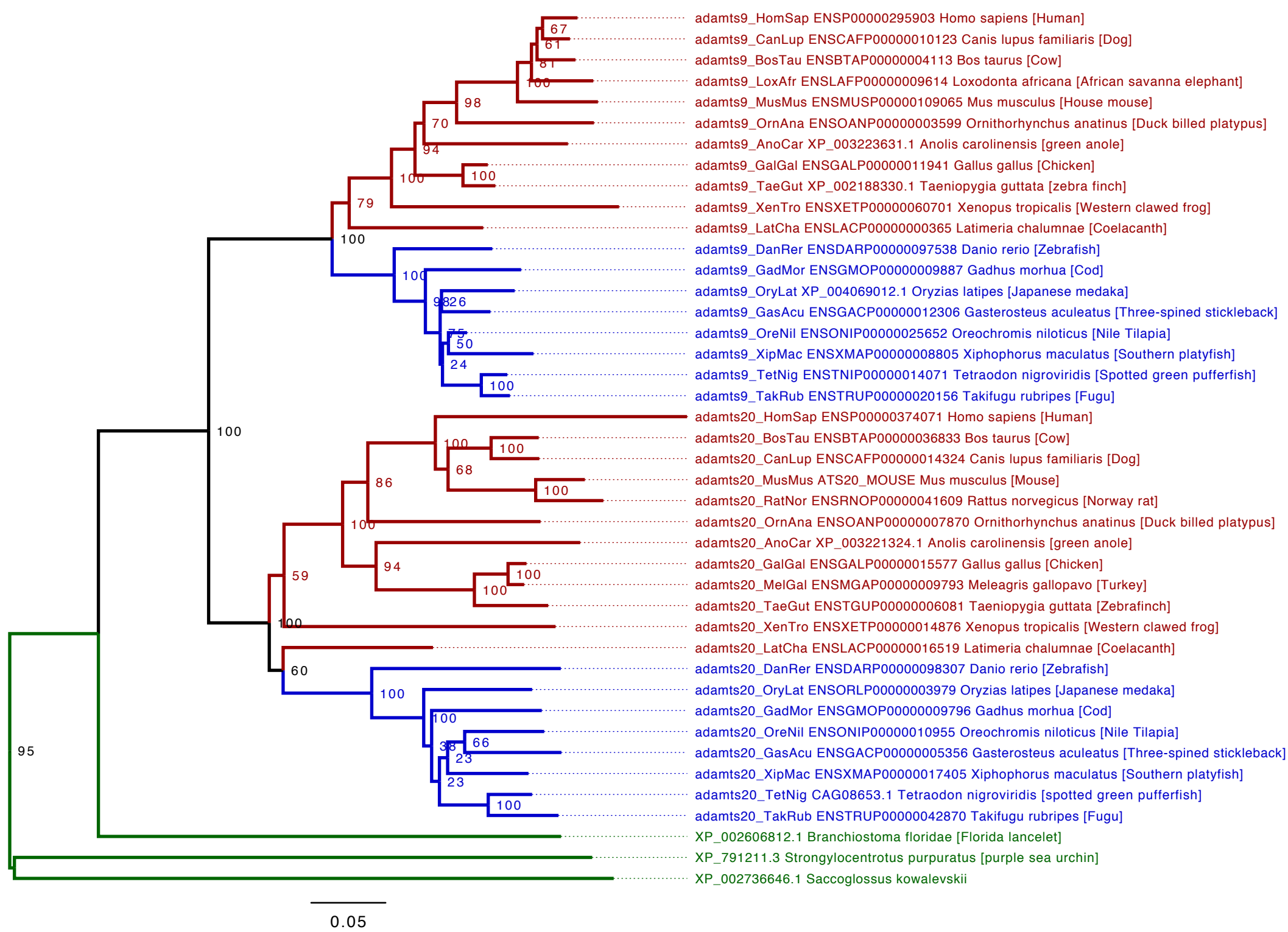

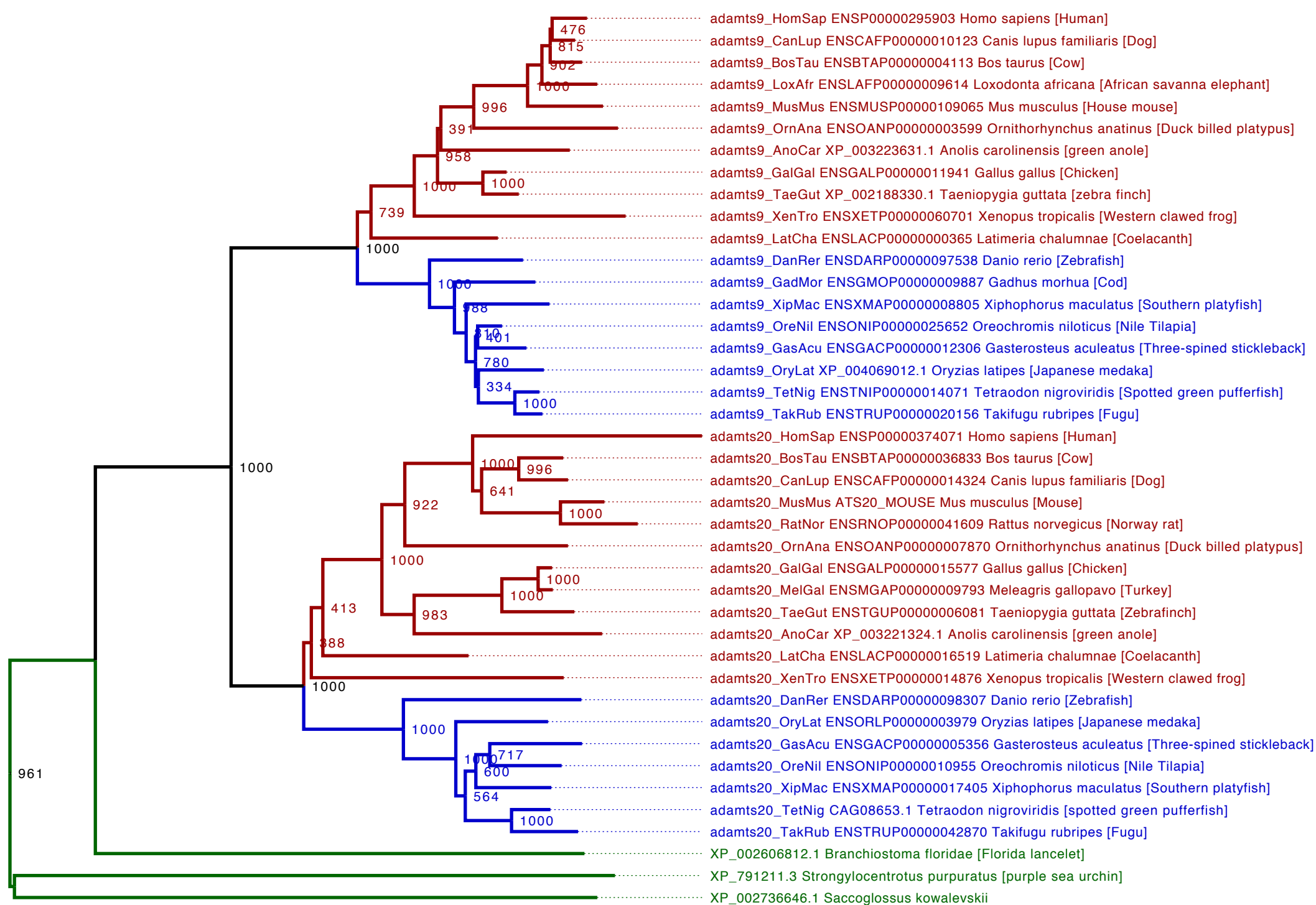

0.06

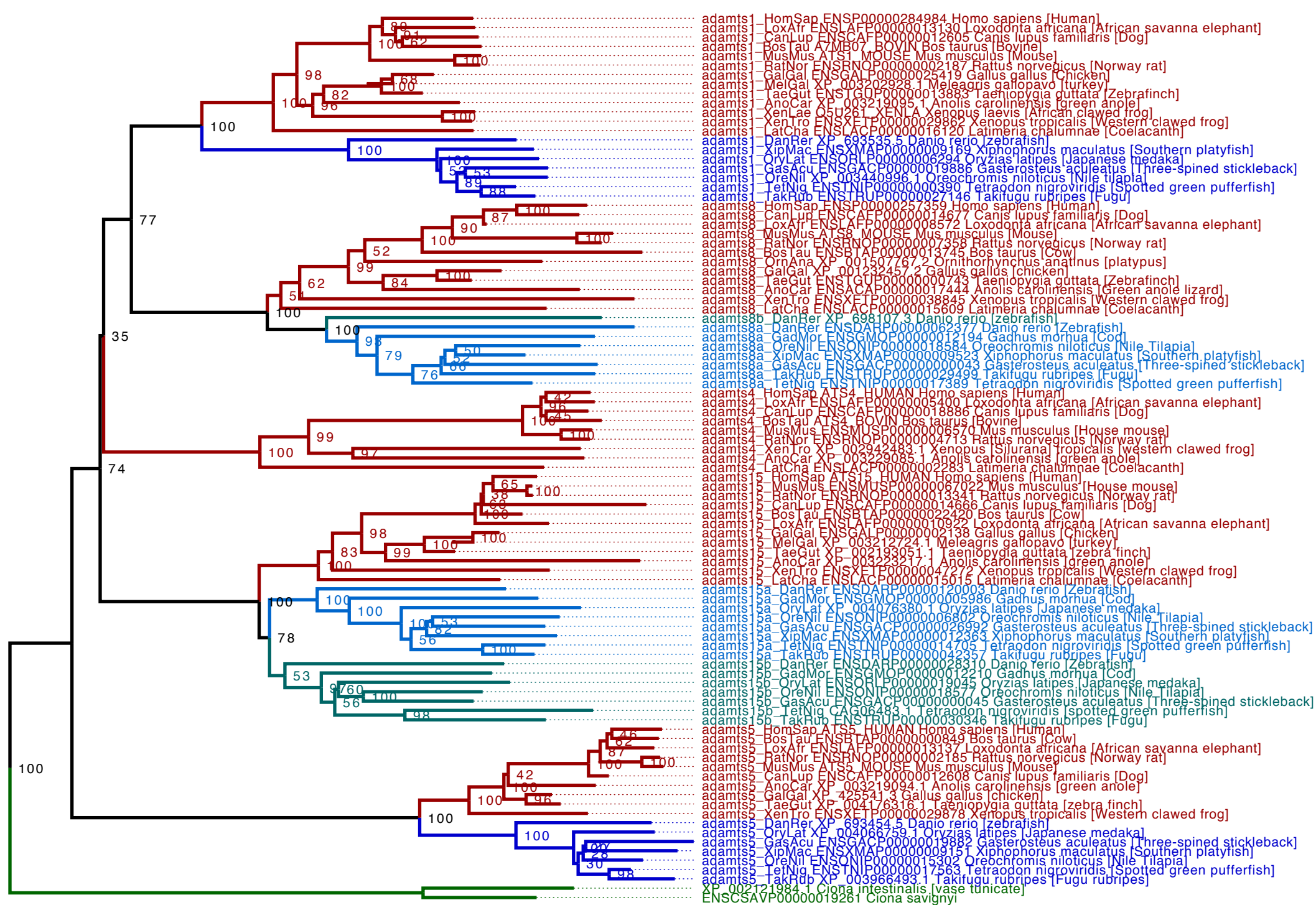

0.06

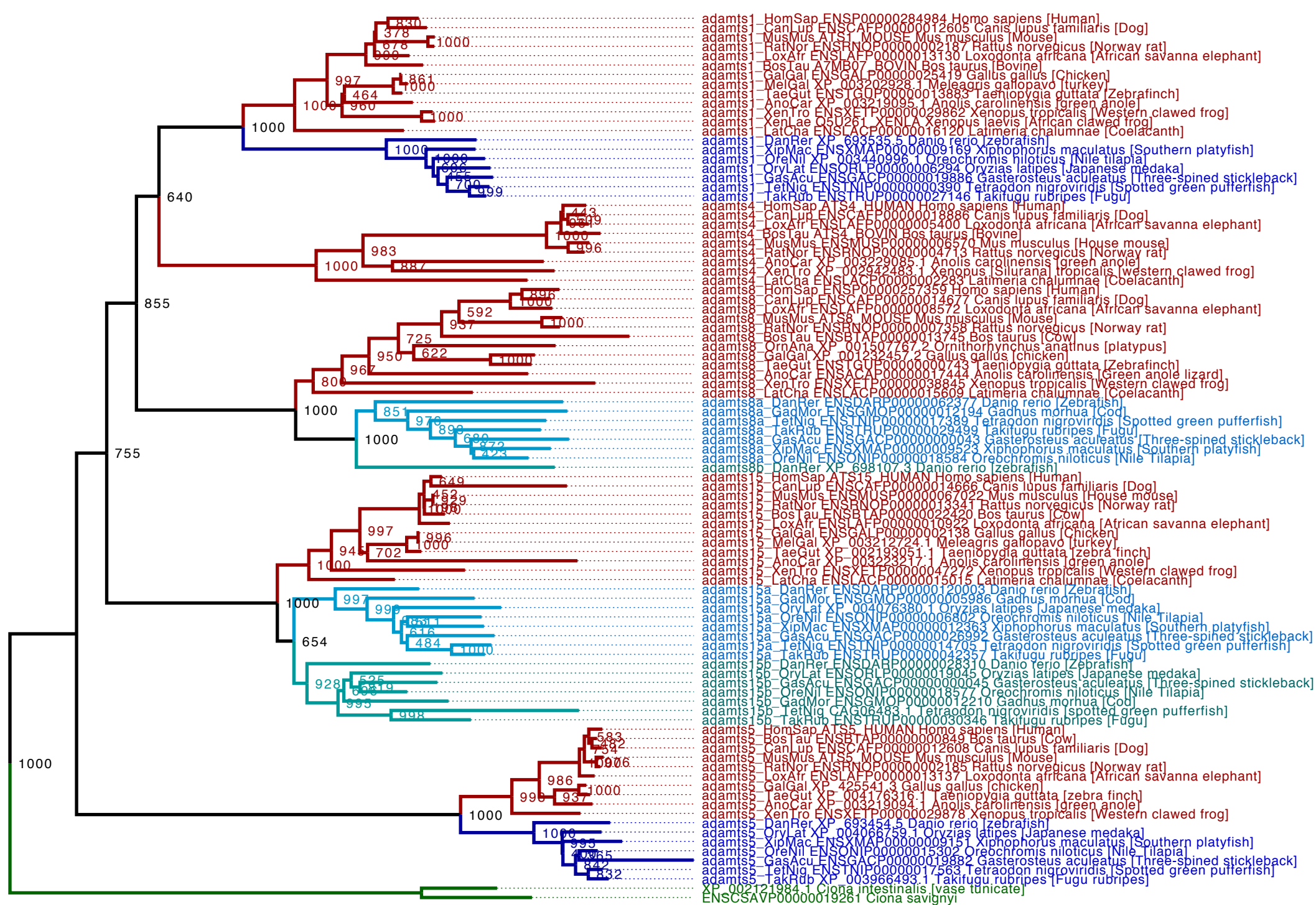

0.07

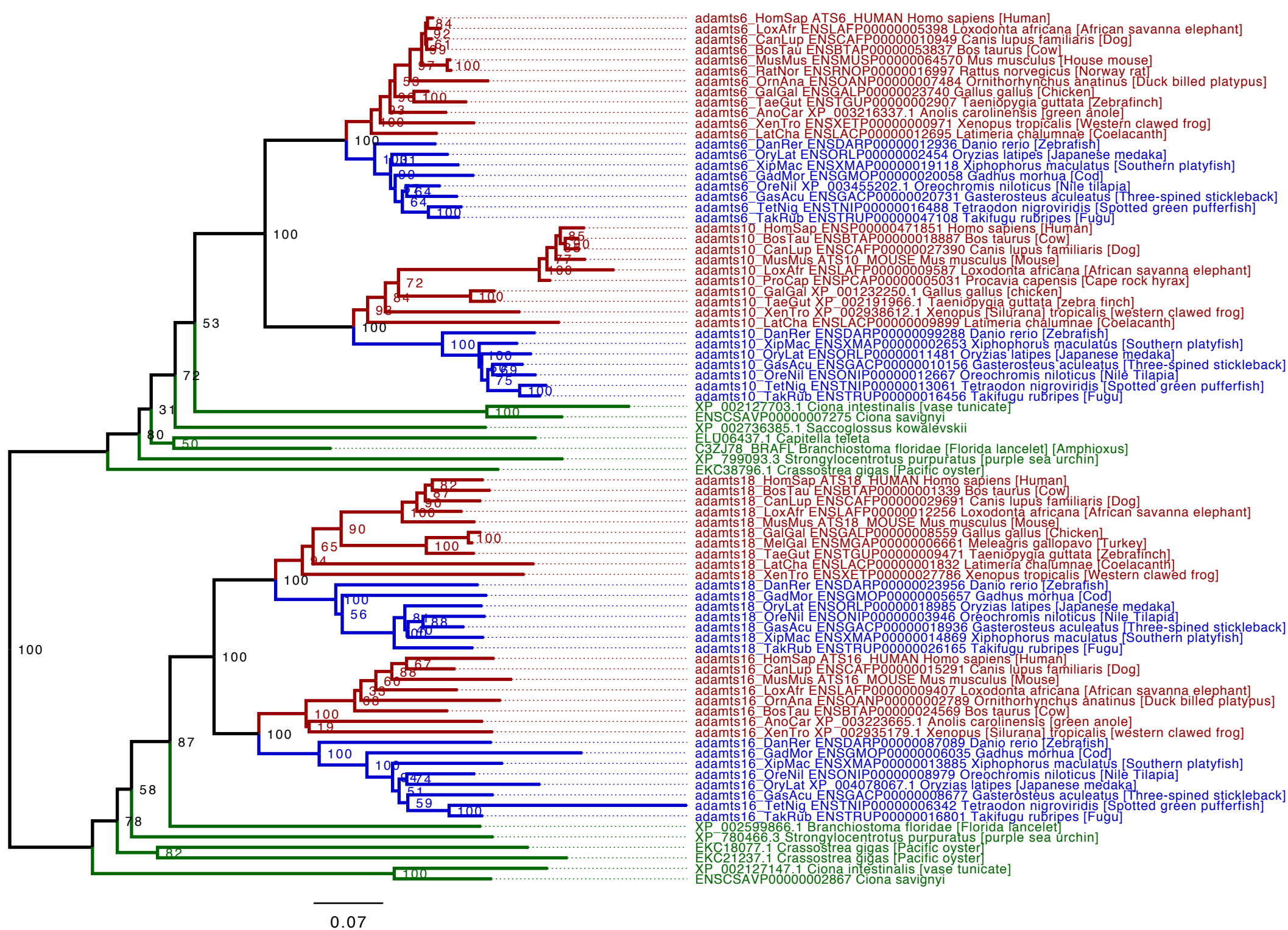

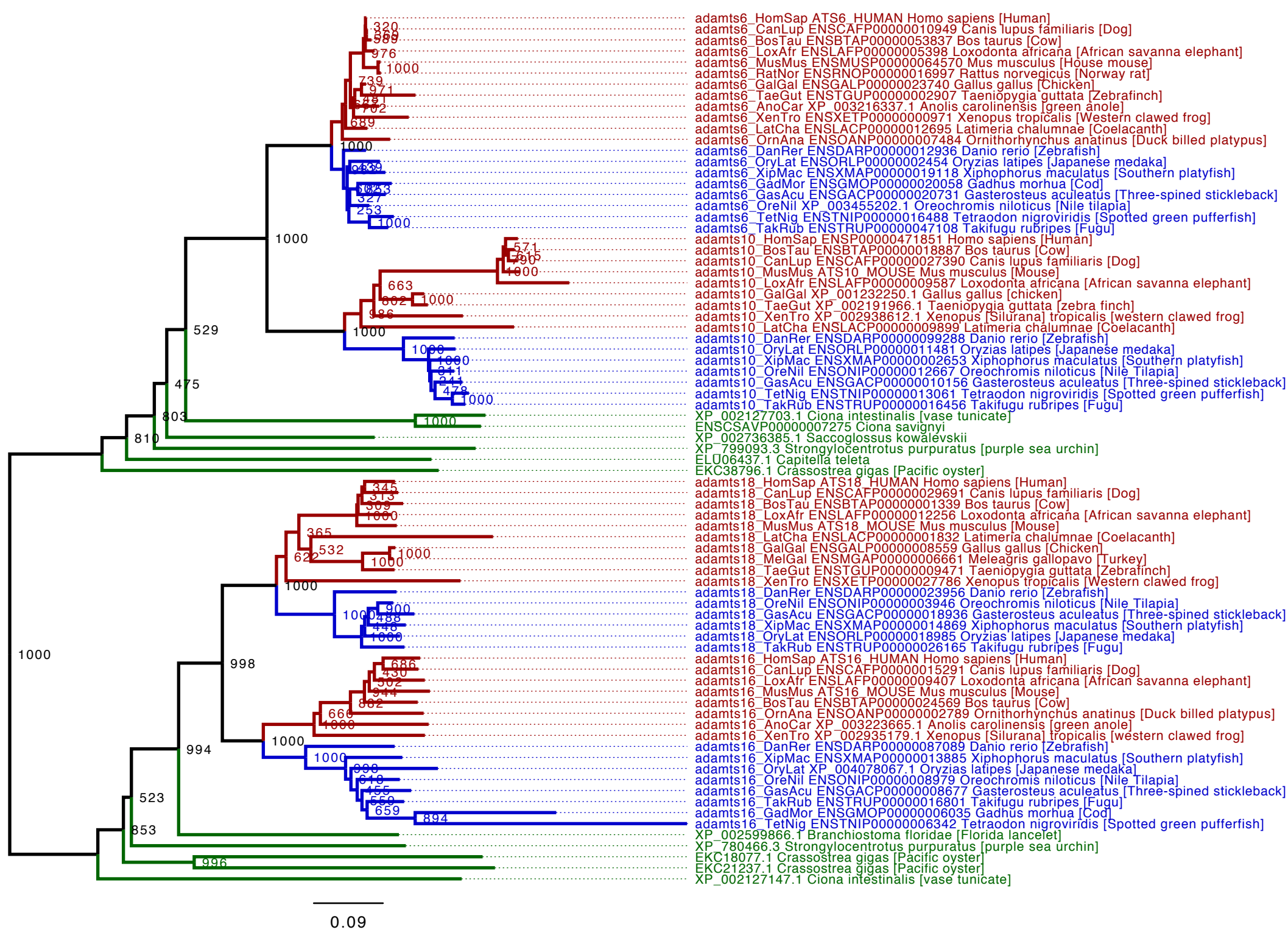

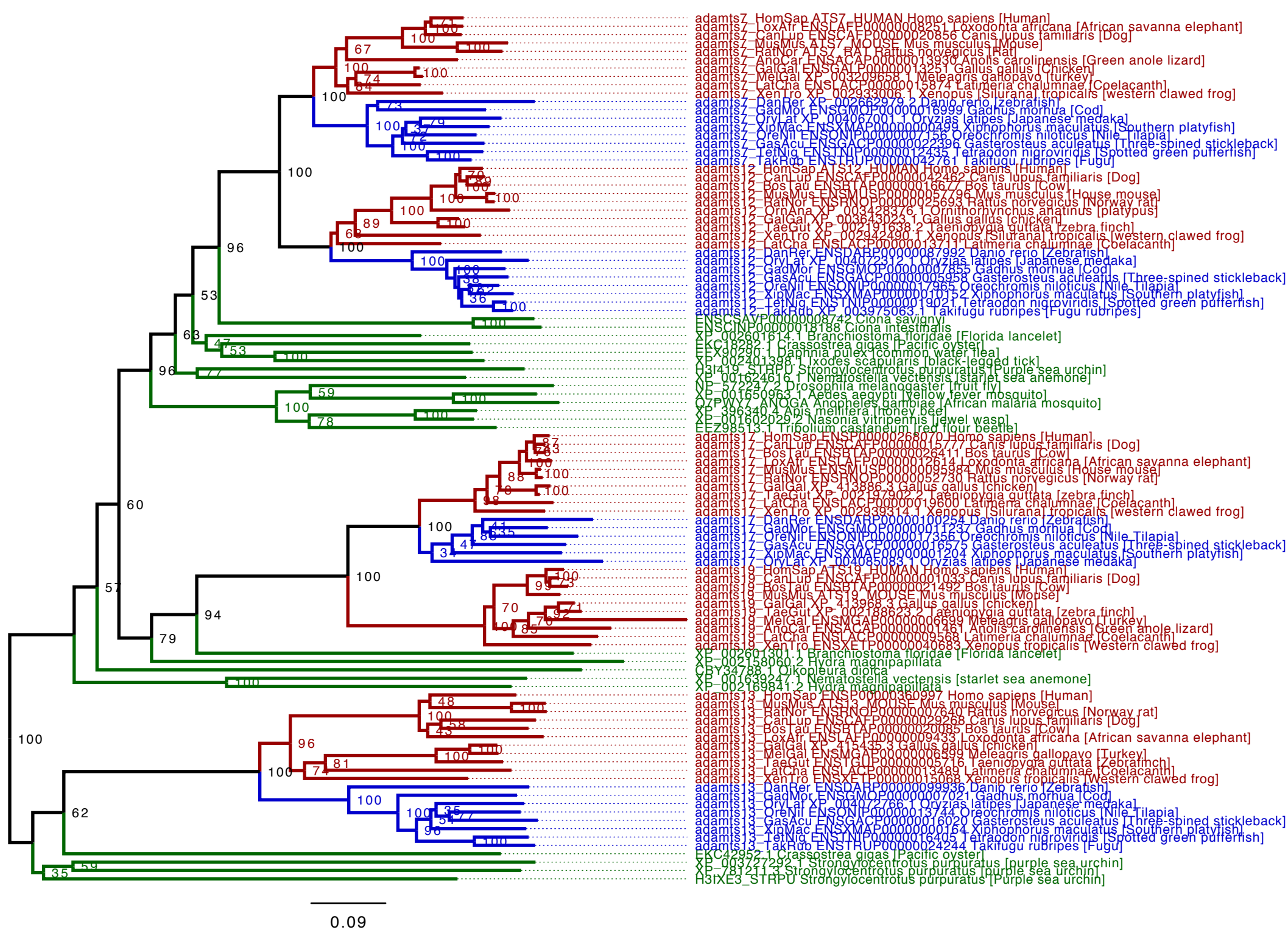

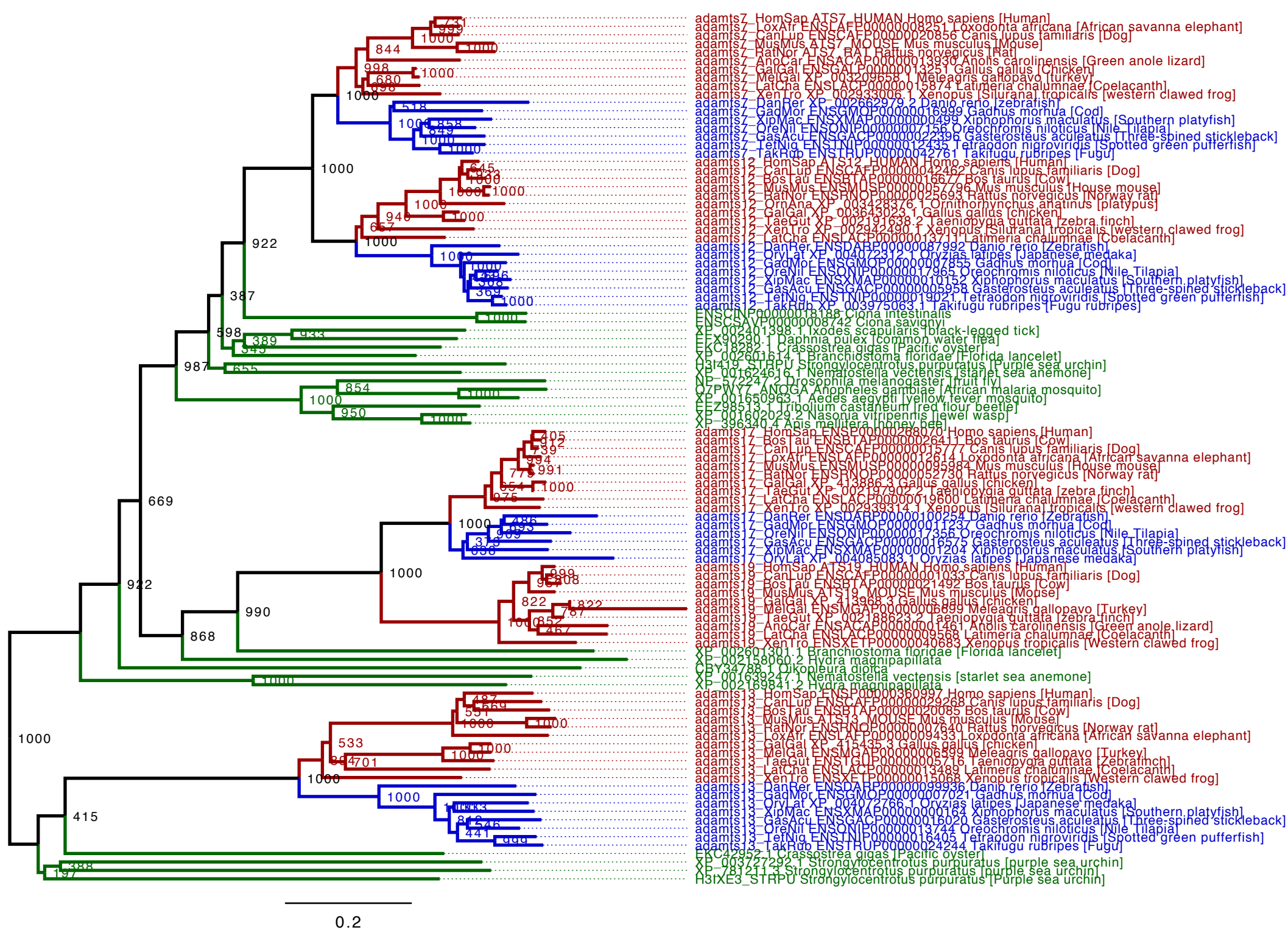

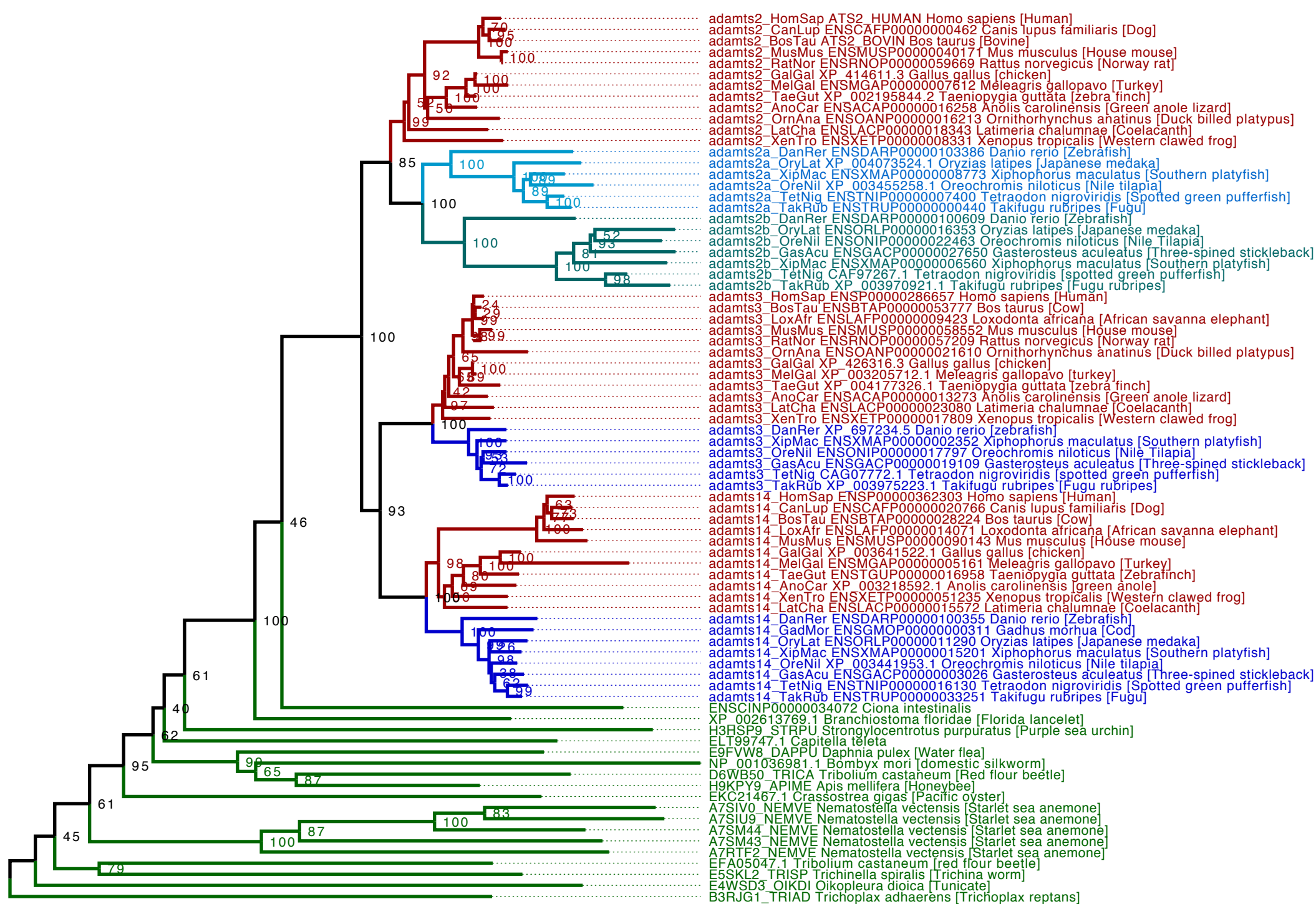

0.1

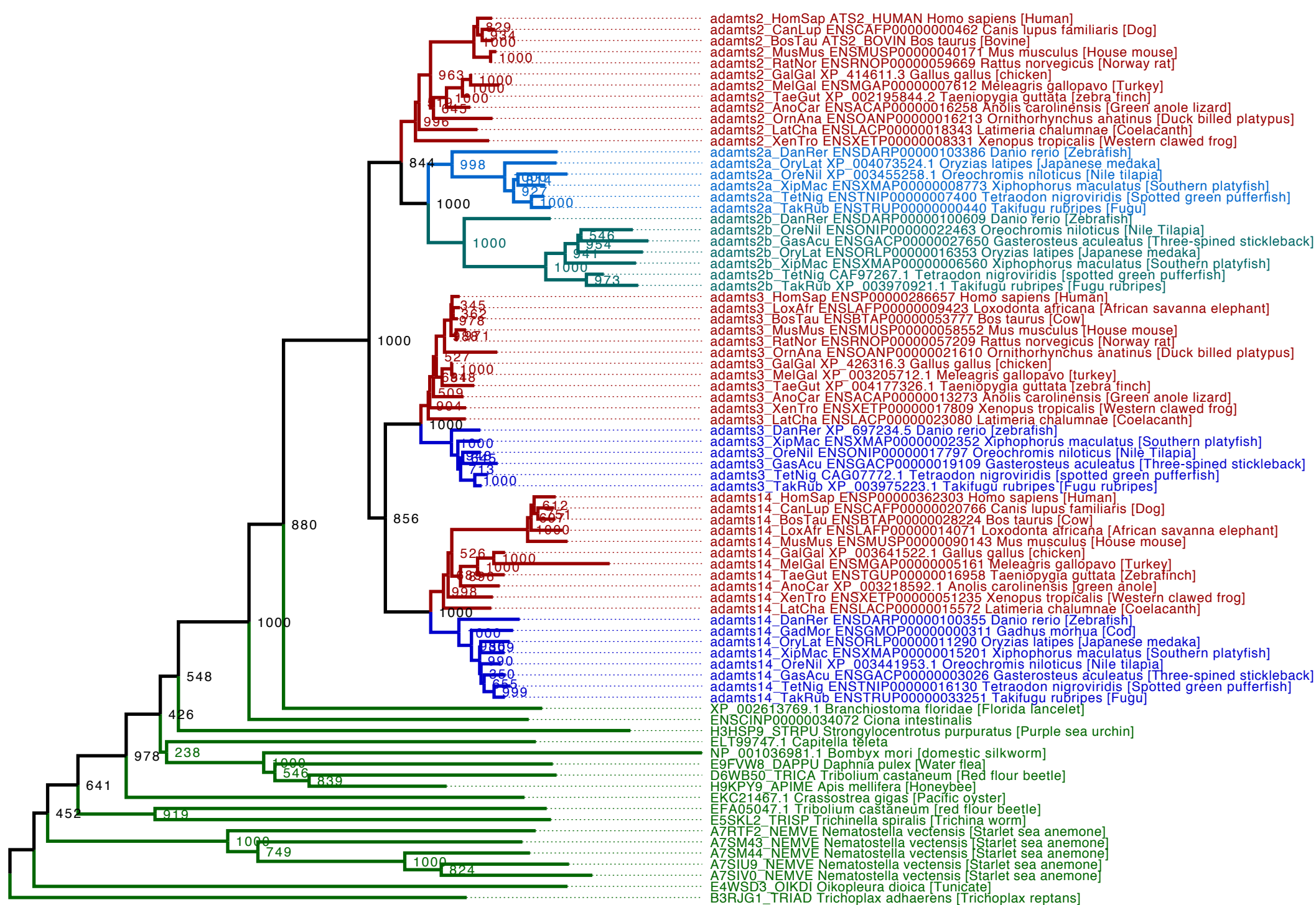

0.2

Supplement: Additional file 2: Figure S2. — Phylogenetic analysis of proteins of the adamts family in vertebrate species. Phylogenetic analyses by maximum likelihood (ML) of all genes of the adamts family based on the largest shared protein sequences. Sarcopterygii are colored in red, Actinopterygii and in blue, two blue colors are used for 3R-WGD duplicated genes in teleosts, non vertebrate species are in green. Protein IDs are attached to the gene name and organism species. [file 12862_2015_281_MOESM2_ESM.pdf]

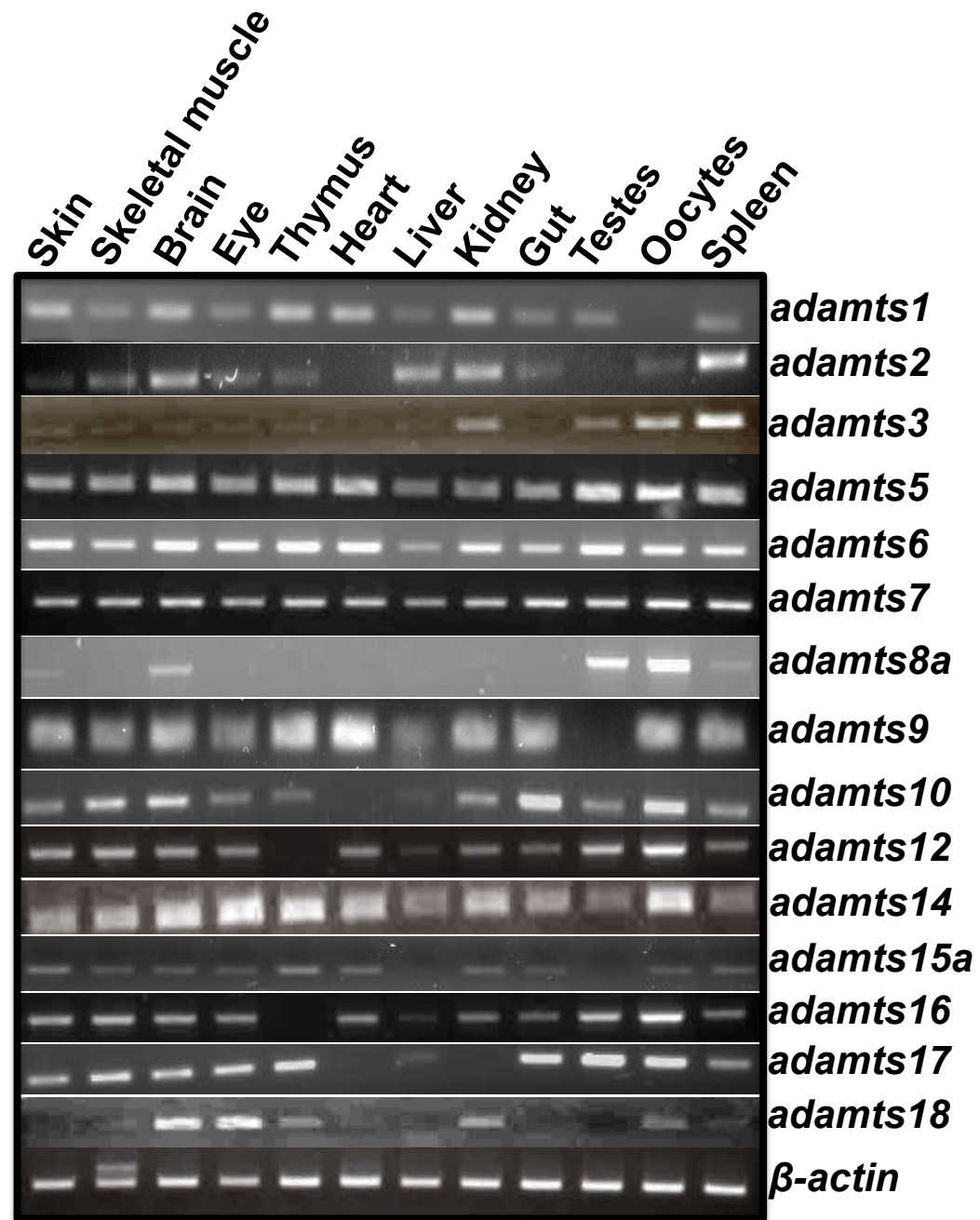

Brunet et al, 2014 Supp. Fig. 4

Supplement: Additional file 4: Figure S4 — Expression of adamts genes in adult zebrafish. RT-PCR of ADAMTS enzymes in adult zebrafish tissues. Total RNA was isolated from adult zebrafish organs and RT-PCR was performed for each adamts enzyme with same primer sets used for quantitative RT-PCR. β- actin was used as a house-keeping gene. [file 12862_2015_281_MOESM4_ESM.pdf]
